# Tweaking the Small Non-Coding RNAs to Improve Desirable Traits in Plant

**DOI:** 10.3390/ijms24043143

**Published:** 2023-02-05

**Authors:** Koushik Halder, Abira Chaudhuri, Malik Z. Abdin, Asis Datta

**Affiliations:** 1 National Institute of Plant Genome Research, Aruna Asaf Ali Marg, New Delhi 110067, India; 2 Centre for Transgenic Plant Development, Department of Biotechnology, School of Chemical and Life Sciences, Jamia Hamdard, New Delhi 110062, India

**Keywords:** small non-coding RNAs, siRNA, miRNA, tasiRNA, abiotic stress

## Abstract

Plant transcriptome contains an enormous amount of non-coding RNAs (ncRNAs) that do not code for proteins but take part in regulating gene expression. Since their discovery in the early 1990s, much research has been conducted to elucidate their function in the gene regulatory network and their involvement in plants’ response to biotic/abiotic stresses. Typically, 20–30 nucleotide-long small ncRNAs are a potential target for plant molecular breeders because of their agricultural importance. This review summarizes the current understanding of three major classes of small ncRNAs: short-interfering RNAs (siRNAs), microRNA (miRNA), and transacting siRNAs (tasiRNAs). Furthermore, their biogenesis, mode of action, and how they have been utilized to improve crop productivity and disease resistance are discussed here.

## 1. Introduction

Two major kingdoms in taxonomy, kingdom Animalia and kingdom Plantae, divide this whole universe, and among these two kingdoms, Plantae is non-motile. Hence, evading stress or constantly dynamic environmental conditions has posed a constant challenge for plants. Avoidance, tolerance, or resistance to abiotic and biotic stress factors can substantially minimize yield loss, which could be a turning point to increase food security for millions. Researchers are seeking solutions and a permanent way out from these harsh and demanding situations; non-coding RNAs (ncRNAs) have come to the rescue. Their dramatic development as a rescue agent among the food and cash crops has emerged as a windfall in the plant science field. These ncRNAs are classified as significant bioactive molecules and are substantial players in generating diverse genotypes and phenotypes. It is a well-established fact that 90% of the genome in eukaryotes is transcribed into mRNA, from which only 2% are translated and finally produce proteins [1,2]. Other than this essential 2%, the rest is made up of ncRNAs. These RNAs were condemned as useless because they could not deliver the signature function of RNA, which is characterized by its protein-coding capacity and also imperfectly conserved sequences [1,3]. Over the years, it has been established experimentally and by high-throughput sequencing that ncRNAs are linked to a plethora of gene regulatory functions categorized as epigenetic, transcriptional, or post-transcriptional fields [4,5,6].

Soon after their discovery, the ncRNAs were labeled into classes based on their origin, biogenesis, and mode of action. There are (a) housekeeping RNAs and (b) regulatory RNAs. The list of regulatory ncRNAs has two members, namely small non-coding RNAs (Table 1), which include microRNAs (miRNAs); short interfering RNAs (siRNAs); piwi-interacting RNAs (piRNAs) (in animal only); transacting siRNA (tasiRNA); and long non-coding RNAs (lncRNAs). This category of RNA follows the usual route of being transcribed from DNA, but they do not carry out the translation process and produce proteins [7]. They generally specialize in performing other crucial activities related to the growth and development of plants and, most essentially, bring about responses to various abiotic stress factors both transcriptionally and post-transcriptionally [8]. 

It has been inferred from modern research that ncRNAs play a vital role in the regulation of gene expression (transcriptional and post-transcriptional) during development and stress response (abiotic and biotic) in plants [9]. In this review, we delve into a detailed discussion about the biogenesis and structure of small ncRNAs (miRNA, siRNA, and tasiRNA) along with an in-depth analysis of their mode of action (how they repress the target gene expression) and about the wide variety of roles choreographed by them in plant gene silencing. 

**Table 1:** Classes of small non-coding RNAs involved in gene silencing in plants.

Class	Mechanism	Length (nt)	References
Short interfering RNA (siRNA)	Targeted mRNA cleavage	21–24	[10]
MicroRNA (miRNA)	Translational silencing, mRNA cleavage	20–24	[11]
Trans-acting siRNA (tasiRNA)	mRNA cleavage	21–22	[12]
Phased secondary small interfering RNAs (phasiRNAs)	mRNA cleavage, function in development	20–21	[13]

## 2. Short Interfering RNAs 

RNA interference (RNAi) has inarguably been a revolutionary discovery in the field of biology in the last several decades. The discovery of small (20–30 nucleotide long) non-coding RNAs that can regulate genes and the genome completely transformed RNA biology. These small RNAs can guide effector proteins targeting any complementary nucleotide sequence through the RNAi pathway, thereby downregulating its expression level. Napoli and Jorgensen first reported small RNA-mediated gene regulation in plants while working with chalcone synthase (CHS) in petunia [14]. It was referred to as co-suppression/ post-transcriptional gene silencing (PTGS). Later in 1992, a similar phenomenon was reported in a fungus (*Neurospora crassa*), which is termed quelling [15], and in *Caenorhabditis elegans* by Guo and Kemphues (sense/anti-sense mRNA) in 1995 [16]. Then, in 1998, Fire and Mello explained for the first time that double-stranded RNAs (dsRNAs) were the actual reason behind such silencing of endogenes while working with *Caenorhabditis elegans* [17]. Andrew Fire and Craig Mello were awarded the prestigious Nobel prize in 2006 in the category of Physiology/Medicine for discovering RNAi: gene silencing by dsRNAs. It has led to extensive research on how small RNAs can regulate several essential aspects of a plant’s life, such as transcription, RNA processing, RNA stability, chromosome segregation, etc. 

### 2.1. Biogenesis of Short Interfering RNAs

**Figure 1:**
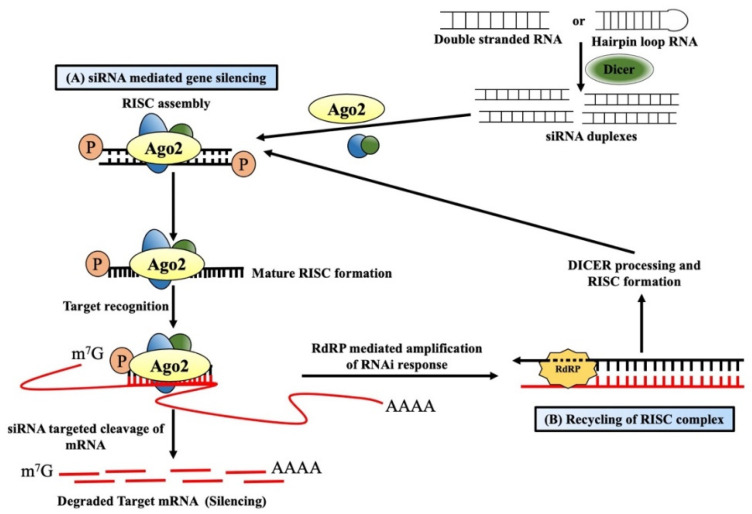
Mechanism of short-interfering RNA-mediated gene silencing. (**A**) Long double-stranded RNAs/hairpin loop RNAs from alien genes are processed into short interfering RNAs (siRNAs) by the Dicer/TRBP (the human immunodeficiency virus transactivating response RNA binding domain) complex and finally become incorporated into RNA-induced silencing complex (RISC). One strand (passenger strand) is degraded from the RNA duplex, and the other strand (guide strand), along with argonaute 2, forms the active RISC. The guide strand guides the active RISC to target and cleaves the complementary mRNAs into the cytosol, resulting in gene silencing. (**B**) RNAi response in plants and worms generally becomes amplified by the RNA-dependent RNA polymerase enzymes (RdRPs). RdRPs and RISC use targeted mRNAs as a template to generate double-stranded RNAs dsRNAs, which then are processed by the Dicer into secondary siRNAs. These siRNAs eventually amplify the RNAi effect in the system.

dsRNA-mediated post-transcriptional gene silencing (PTGS) symbolizes the cellular defense mechanism protecting it from foreign nucleic acids of invading viruses or transposons [18,19]. Such integration of alien genes produces dsRNAs, which act as a guide for sequence-specific RNA degradation and are believed to be associated with maintaining the silencing process for a long time [20,21]. The processing of these long dsRNAs into 21–24 nt long short-interfering RNAs (siRNAs) is facilitated by Dicer, a ribonuclease III enzyme [22] (Figure 1A). The presence of these siRNAs was first reported by Hamilton and Baulcombe in plant tissues that showed virus-induced PTGS [23]. These siRNAs were later found to be present also in *Drosophila melanogaster* embryo lysate [24], where the added synthetic 20–22 nt RNA duplexes can efficiently target and cleave mRNAs at 21 nt intervals [25]. That is why these 21 nt long RNAs were called siRNAs or silencing RNAs. Dicer-mediated processing is coupled with other co-factors, and these siRNAs are finally loaded onto the RNA-induced silencing complex (RISC), a member of the argonaute protein family [26]. During incorporation within the RISC, one strand called the “passenger strand” is dissociated by the activity of AGO2, which is encoded by the gene *AGO2* (argonaute RISC catalytic component 2), whereas the other “guide strand” that serves as a guide for RNA-directed sequence-specific silencing stays within the complex, forming the mature RISC [27,28]. Targeted mRNA with sequence complementarity with the 21 nt long guide-siRNA within the mature RISC is cleaved between the 10th and 11th nucleotide (from the 5′ end) by the PIWI domain of the AGO2 protein, generating products containing 5′-monophosphate and 3′-hydroxyl termini [29,30]. These cleaved products are rapidly degraded by the endogenous exonuclease activity due to the lack of 5′ capping or a 3′poly(A) tail [31]. Apart from the above-mentioned RNAi, plants [20,32], fungi [33], and *C. elegans* [21,34] possess a special enzyme called RNA-dependent RNA polymerases (RdRPs), which can produce additional dsRNAs for amplifying the RNAi response. Such dsRNAs are synthesized in a primer-independent manner using the targeted mRNA as a template. It is subsequently processed by Dicer to produce more siRNAs, thereby facilitating the recycling of the RISC complex [35] (Figure 1B). 

### 2.2. RNA-Induced Silencing Complex: The Versatile Gene Silencing Complex

Although there are diverse ways to regulate gene expressions using RISC, two significant incidents are common for all types. Firstly, every RISC should comprise one argonaute protein family member, and secondly, at its core, a small RNA should guide the RISC to target mRNAs through Watson–Crick base pairing [36]. Each class of small RNAs in eukaryotes, such as siRNA/miRNA/piRNA (only in animals), together with the AGO protein family, forms the ribonucleoprotein RISC. Our current understanding of RISC (from birth to death) has been summarized here. 

**Figure 2:**
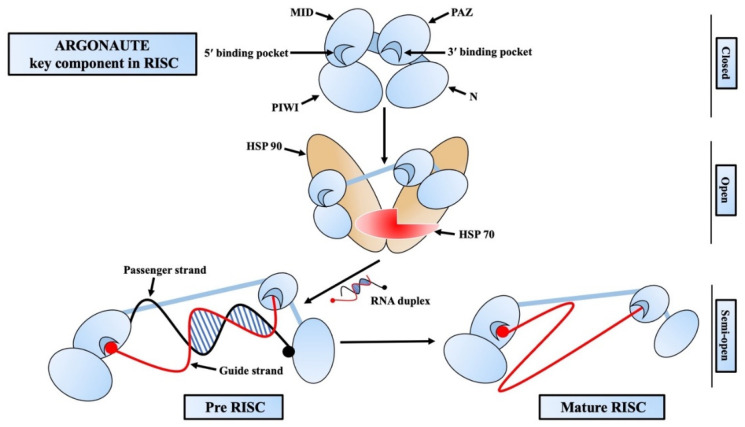
Mechanism of RNA-induced silencing complex (RISC) assembly. Argonaute has four functional domains: PIWI-AGO-Zwille (PAZ), Middle (MID), N-terminal (N), and PIWI (Piwi/Argonaute/Zwille). An empty argonaute loads an RNA duplex with the help of HSP70/HSP90 and forms the pre-RISC. The passenger strand (black strand) is ejected from the pre-RISC, and the guide strand (red strand) and argonaute form the mature/active RISC that start targeting the complimentary mRNAs to assert gene silencing.

The Argonaute family of proteins is at the heart of RISC-mediated gene regulation [37,38]. There are four functional domains in AGO proteins: PIWI-AGO-Zwille (PAZ), Middle (MID), N-terminal (N), and PIWI (Figure 2) [37,39]. There are two linkers (L1, L2), out of which L1 connects the N and PAZ domains, whereas L2 supports the N-L1-PAZ structure connecting it to the MID-PIWI lobe [40,41]. The PAZ domain of AGO proteins contains a pocket that interacts with and binds the guide strand from the 3′ end [42]. A conserved sequence of a catalytic tetrad (Asp-Glu-Asp-His/Asp) can be found in the PIWI domain of some AGO proteins that are responsible for the target mRNA degradation. This catalytic domain is also responsible for the cleavage of some passenger strands before it gets ejected [39]. In the initial phase of RISC assembly, a small RNA duplex is loaded onto an empty AGO protein with the help of Hsp70/Hsp90 chaperons and forms the pre-RISC [43]. One of the strands, which is less stable and likely to be adenine/uridine (AU) rich, is preferably able to be the guide strand. This type of strand selection is asymmetric and generally depends on the difference in the thermodynamic stability of two ends of the RNA duplex [44,45]. At the initial stage of passenger strand separation, the N domain disrupts the 3′ end base-pairing of the guide strand to help open the RNA duplex [46]. Then, the passenger strand is sliced at a position opposite the guide strand’s 10th and 11th nucleotide (g10 and g11) by the catalytic activity of the AGO protein [27,28,47]. After passenger strand separation, the mature RISC binds to the targeted mRNA guided by the guide strand and either slices it directly or induces translational repression by recruiting necessary proteins [48,49]. Although AGO and small RNAs are short-lived, once the RISC is formed, those two tend to be long-lived [50,51]. Generally blank/unloaded AGO proteins are degraded by the autophagy pathway [52,53], whereas the target RNA-directed miRNA degradation (TDMD) targeted RISC are degraded by the ubiquitin–protease system [54,55].

### 2.3. Short Interfering RNA Mediated Silencing in Plants

The siRNA-mediated gene silencing mechanism has been exploited extensively over the years for crop protection against biotic stresses and as a platform for overall crop improvement. RNAi-mediated silencing requires the design of a hairpin loop structure containing both the sense and anti-sense strand of the targeted gene separated by an intron sequence. Such a construct will create hpRNAs in plants that are cleaved by Dicer and form siRNAs, which trigger the RNAi pathway to silence the targeted gene [56]. Pests such as insects, nematodes, viruses, and bacteria pose a severe threat to agricultural produce. RNAi has been used to make pest-resistant crops by targeting essential genes in the pests and silencing them by host plant-mediated RNAi (host-induced gene silencing—HIGS) [57].

The two-spotted spider mite is one of the deadliest plant pests; it can attack more than three thousand crops and feeds mainly on Chinese cabbage. RNAi-mediated targeting against the *COPB2* gene of this pest resulted in an almost 100% mortality rate [58]. Similarly, transgenic potato plants expressing the molting-associated *EcR* gene showed enhanced resistance against the deadly Colorado potato beetle [59]. The root-knot nematode *Meloidogyne incognita* causes colossal damage to agricultural products worldwide. RNAi-mediated simultaneous silencing of *Mi-flp1*, *Mi-flp12*, and *Mi-flp18* genes resulted in enhanced resistance against this nematode [60]. An extensive list of such siRNA-mediated gene silencing to improve desirable traits in plants is listed in Table 2. Because RNAi-derived plants have been categorized as genetically modified crops (GM crops) and have been the subject of bitter controversy, the exogenous application of RNAi-inducing dsRNA-based bio-pesticides is gaining popularity, as it provides a non-transgenic approach [61]. Tenllado et al. first reported the effective foliar application of dsRNAs targeting the Alfalfa mosaic virus (AMV), Tobacco etch virus (TEV), and Pepper mild mottle virus (PMMoV) in 2001 [62]. However, the authors did mention that the commercial success of such topical application of dsRNAs will depend on two critical parameters: cost-effectiveness and an optimized mode of delivery. In recent years, several studies have been conducted to deliver such topical solutions of dsRNAs with impeccable efficiency and target specificity [62,63,64]. Topically applied dsRNA-based biopesticides have high species specificity, low levels of toxicity, and a minimal environmental effect compared to traditional pesticides. If their distribution and usage can be regulated in a precautionary way, dsRNA-based biopesticides can revolutionize the integrated pest-management system [65]. 

### 2.4. Tweaking the siRNAs to Improve Desirable Traits in Plants

siRNAs are well known for their silencing role in the case of viral RNAs. They play a master role in regulating plant defense machinery against potential pathogens such as bacteria, viruses, fungi, oomycetes, and other parasitic plants. Recent research has provided evidence of the ability of siRNAs to suppress fungus and oomycetes by silencing specific pathogen genes related to pathogenesis. Thus, scientists have precisely concluded that siRNAs are a potential concoction of diverse gene sequences and are used as a “shotgun” which targets random genes of the pathogen with great efficiency [66]. After discovering the antiviral factor, virus-derived siRNAs (vsiRNAs) in tobacco infected with potato virus, there has been a breakthrough in plant immunity. These viral dsRNAs are directly targeted by plant DCLs, resulting in 21–24 nt long primary vsiRNAs. It has been established that these 21 nt long vsiRNAs specifically silence the detrimental viral RNAs through Post Transcriptional Gene Silencing (PTGS) [67]. The functions of vsiRNAs can also be categorized into two parts. One part is when the vsiRNAs degrade the intruder viral genome and render antiviral tolerance to plants. On the other side, some vsiRNAs silence host gene expression and manipulate host resistance towards viral attack. During tomato yellow leaf curl virus (TYLCV) infection, it has been observed that vsiRNAs are utilized by TYLCV to silence *SILNR1*, a long noncoding RNA (lncRNA) associated with antiviral defense. vsiRNAs obtained from wheat yellow mosaic virus (WYMV) down-regulate host genes and activated broad-spectrum host immunity [68]. Another category of siRNA, which was first seen in Arabidopsis, which also participates in antiviral defense, is virus-activated siRNA (vasiRNA) [69]. In the case of antiviral defense, the vsiRNAs are generated from the viral genome, which safeguards the plants by destroying the viral RNA. However, in the case of non-viral plant pathogens, endogenous siRNA-orchestrated gene silencing is instantly triggered to alter the gene expression associated with plant immunity [66]. The components of the siRNA pathway interact among themselves and others to orchestrate plant immunity. RDR6 is essential for plant immunity because it aids in producing secondary siRNAs and silencing signals. For example, in rice, *shl2-rol*, which is a mutant line of the rice gene *OsRDR6* results in severe infection symptoms when the plant is attacked by *Xanthomonas oryzae* PV. *oryzae*, thus proving the importance of RDR6-dependent siRNAs in rendering tolerance against bacteria [70]. 

Artificial siRNAs are produced in plants via Host-Induced Gene Silencing (HIGS) to silence the deadly pathogen genes causing infection. A well-tested example is transgenic barley and wheat, which express artificial siRNAs that target gene *Avra10* and display increased resistance to *Blumeria graminis*, the causative agent of powdery mildew disease [71]. HIGS via engineered dsRNA resists parasitic plants. This strategy has been demonstrated in transgenic tobacco that expresses dsRNA against transcription factors controlling haustoria development and reduced vigor in *Cuscuta patagonia*. Similarly, *Orobanche aegyptiaca* has been recorded to grow and feed on tomatoes. The above examples highlight host-produced synthetic siRNAs’ significant role in controlling parasitic plants. An extensive list of such siRNA-mediated gene silencing to improve desirable traits in plants is listed in Table 2.

**Table 2:** Genetic alteration of siRNAs in plants to improve the desirable traits.

Plant	Targeted Gene	Traits Involved	References
Wheat	*SBE IIa* and *SBE IIb*	Increased amylose content	[72]
Rice	*OsDWARF4*	Improved biomass	[73]
Cotton	*Δ 9-desaturase* and *oleoyl- phosphatidylcholine γ6-desaturase*	Improved stearic- and oleic- fatty acid content	[74]
Onion	*Lachrymatory factor synthase (LFS)*	Tearless onion	[75]
Rice	*OsGA20ox2*	Improved grain yield	[76]
Cotton	*Delta-cadinene synthase*	Reduced toxic terpenoid gossypol	[77]
Rice	*OsSSI2*	Resistance against *Magnaporthe grisea* and *Xanthomonas oryzae*	[78]
Rice	*GluB*	Decreased glutelin content	[79]
Rice	*OsFAD7* and *OsFAD8*	Resistance against *Magnaporthe grisea*	[80]
Tomato	*SlNCED1*	Increased concentration of β-Carotene and lycopene	[81]
Potato	*SYR1*	Resistance against *Phytophthora infestans*	[82]
Tomato	*DET1*	Increased concentration of Carotenoid and flavonoid	[83]
Wheat	*MLO*	Resistance against *Blumeria graminis f.* sp. *tritici*	[84]
Tomato	*α-Man/β-Hex*	Increased fruit shelf-life	[85]
Arabidopsis	*16D10*	Resistance against *Meloidogyne incognita*	[86]
Tomato	*ACC synthase (ACS)*	Decreased content of ethylene	[87]
Tobacco	Splicing factor and integrase	Resistance against *Meloidogyne incognita*	[88]
Tomato	*Chalcone synthase*	Production of Seedless fruit	[89]
Rice	*PNS12*	Resistance against Rice Dwarf Virus (RDV)	[90]
Arabidopsis	*HC-Pro*	Turnip Mosaic Virus (TuMV)	[91]
Canola	*Farnesyl transferase*	Increased drought tolerance	[92]
Rice	*OsDIS1*	Increased drought tolerance	[93]
Sugarcane	*CP*	Enhanced resistance against sugarcane mosaic virus	[94]
Potato	*eIF4E*	Enhanced resistance against potato virus Y	[95]
Soybean	*AC2*	Improved resistance against mungbean yellow mosaic India virus	[96]
Soybean	*CP*	Improved resistance against mungbean yellow mosaic India virus	[97]
Soybean	P3 cistron	Enhanced resistance to soybean mosaic virus	[98]
Rice	*S7-2*	Improved resistance against rice black streak dwarf virus	[99]
Cotton	*IR*	Improved resistance against cotton leaf curl Rajasthan virus	[100]

## 3. microRNA: Structure and Biogenesis 

MicroRNAs are extremely well-known members of molecular biology as a group of tiny 20–24 nt (as opposed to siRNA, Table 3) endogenous and non-coding RNAs that mastermind the regulation of gene expression [101]. Researchers worldwide have investigated plants and proved a labyrinthine molecular pathway regarding miRNA biogenesis and their various modes of action. The hub of miRNA biogenesis and activity are clandestine subcellular locations that have been established after decades of research [102]. 

**Figure 3:**
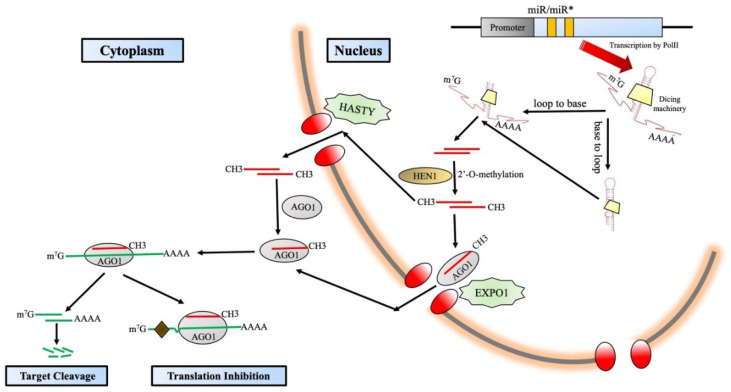
RNA Polymerase II (Pol II) transcribes *MIR*(micro-RNA) genes and forms stem–loop structure by folding to form pri-miRNAs. These are processed by DCL1 either in a “base-to-loop” or “loop-to-base” track. Small RNA methyltransferase HEN1 methylates the newly formed miRNA/miRNA*duplexes. RISC takes place either in the nucleus or cytoplasm. The basic mode of action of miRNAs (gene silencing) takes place via target cleavage or translation inhibition.

A multitude of *MIRNA* genes (*MIR* genes) are encoded by the plant genome, and it has been observed that a vast majority of them survive as clusters [103,104]. There is a plethora of complexities and regulations throughout the miRNA biogenesis pathway [102] (Figure 3). miRNAs have been classified based on their position inside the genome and are “intronic” or “intergenic.” The intronic miRNAs are prepared from the introns present in the host transcript [104]. On the other hand, the intergenic miRNAs connect two protein-coding genes, and their transcription occurs in separate independent units by RNA polymerase II (Pol II). Because they are certified Pol II products, the primary transcripts of the *MIR* genes, also called the pri-miRNAs, have a typical 5′ capping and a 3′ Poly A tail with splicing [105]. Subsequent folding of the pri-miRNAs takes place, which results in a hairpin-like structure comprising an upper stem, a terminal loop, the miRNA zone, a lower stem portion, and a number of arms that are perceived, handled, and fixed by Dicer-like RNase III endonucleases (DCLs). The number of DCL proteins varies across the immense range of plants. For example, in *Arabidopsis thaliana*, the number of DCL proteins is four. DCL1 is the leader protein, and they expedite the generation of the majority of miRNAs with the aid of accessory proteins such as double-stranded RNA-binding protein Hyponastic Leaves 1 (HYL1) along with Serrate (SE), which is a zinc finger protein [106]. Many other DCLs are also engaged in the production of miRNA; for example, miR822 and miR839 are produced by AtDCL4, and OsDCL3a is responsible for producing 24-nt long miRNAs that administer DNA methylation, e.g., hc-siRNAs [107]. In the process of miRNA biogenesis, there are multiple other factors that participate in the process, such as THO2, SE, and RCF3. They create structures called splicing speckles and stay adjacent to dicing bodies [108,109]. Other factors such as MOS2, PINP1, and NOT2 display scattered nucleoplasmic design, and they too remain partially abutting the dicing bodies [110,111,112]. The detailed analysis of the subcellular localization of all the above-discussed factors is highly suggestive that they actively participate in miRNA biogenesis [102]. A remarkably noteworthy feature that has unfolded from the work of [102] is that the pri-miRNAs are processed, folded, and finally modified co-transcriptionally. This concept was hypothesized earlier, but the above studies act as solid evidence for this fact. Firstly, DCL1 is closely connected with the *MIR* genes via their chromatin region [113]. Secondly, the plethora of regulatory proteins (NOT2, CDC5, and ELP2) play a vital role in miRNA transcription and link up with the miRNA machinery functioning proteins [114]. mRNA adenosine methylase (MTA) layers and coats m^6^A on the surface of pri-miRNAs and might also have a profound impact on miRNA biogenesis. In plants, the structure of the pri-miRNAs is comparatively variable in terms of length, which starts from 60nt and might stretch over to 500 nt. In terms of structure, they display more complexity compared to their animal counterparts (~70 nt long) [114], and they can be fixed in two ways: the loop-proximal site to the loop-distal site and conversely as well [115,116,117,118,119,120,121]. 

The DCL-umpired fixation of the nascent miRNA/miRNA* duplex has 2-nt 3′ overhangs on both strands with a phosphate group at the 5′ end and two hydroxyl groups at the 3′ end. These two hydroxyl groups are of extreme necessity, and the 2′-OH site undergoes methylation orchestrated by small RNA methyltransferase HUA Enhancer (HEN1) [122,123]. For years, the prejudice existed that methylated miRNA/miRNA* duplexes were transported out by the animal Exportin 5 (EXPO5) homologue hasty (HST) protein [124]. The assembly of RISC was an enigma to scientists for decades. Later, in 2018, Bologna et al. established the fact that RISC is predominantly assembled in the nucleus and then exported out to the cytoplasm by the EXPO1 protein. Some more research also throws light on the fact that some miRNAs might be exported out of the nucleus in their original duplex form and fabricated in the cytosol altogether. The guide strand, miRNA of the miRNA/miRNA*complex, is fabricated inside the argonaute (AGO) protein, and the passenger strand (miRNA*) is degraded. In *Arabidopsis thaliana*, 10 AGO proteins are present, and AGO1 is the key performer for miRNAs and is designated the chief effector protein [125]. 

**Table 3:** Major differences between siRNAs and miRNAs in plants.

Properties	siRNA	miRNA	References
Discovery	1999	1993	[29,126]
Definition	Cell’s defense mechanism against foreign nucleic acids	Regulator of endogenous genes	[127]
Length	21–24 nt	20–22 nt	[128]
Precursor	Long dsRNAs	Hairpin-shaped ssRNAs	[129]
Gene regulation mechanism	Transcriptional and post-transcriptional	Post-transcriptional only	[128]
Mode of action	Histone modification, DNA methylation, and mRNA degradation	Translational repression and mRNA degradation	[128]
Argonaute requirement	AGO1, AGO4, AGO6, AGO7	AGO1, AGO10	[130,131]
Nature of complementation with target	Fully complementary	Fully/partially complementary	[127]
Functions	Defense against viruses, transposons	Biotic/abiotic stress response, cell development, and differentiation	[128,132,133]

miRNAs mediate gene silencing by guiding the RISC complex to attack the target genes through complementary base pairing, but the primary route they follow is that of either target cleavage or/and inhibition of translation. With extensive research by researchers globally, it has been established that some miRNAs (miR390, miR173, and miR845) have the potential to commence the mass manufacture of secondary siRNAs called the phasiRNAs which also are known as easiRNAs [134,135,136]. In plants, an extremely inflexible base-pairing rule is engaged. It has been observed that an almost impeccable base-pairing happens in the 5′ region, with a maximum mismatch number being one, and a less stringent pairing happens in the 3′ end, with a maximum of four mismatches and very small bulges [137,138]. In plants, the target gene enormity is, to a large extent, lesser than what is found in animals. Apparently, translation inhibition seems to be the most widely accepted route, but practically, target cleavage holds immense importance because of its mandatory participation in the post-germination development of the plant [139].

### 3.1. Mode of Action of microRNA

The elemental phenomenon which dictates miRNA activity is its export from the nucleus to the cytoplasm [105]. In the model plant, *Arabidopsis thaliana* duplexes of miRNA/miRNA* are inferred to be cut off from the pri-miRNAs inside the nucleus itself because the activity of DCL1 is nucleus-specific, and the shifting from the nucleus to the cytoplasm is carried out by HASTY [124]. There is also another functional complex called the THO/TREX complex, which plays some role in miRNA biogenesis [108]. The above-mentioned complex is the mediator of the transcription-coupled export of mRNA through the nuclear pore complex, so scientists have inferred that the THO/TREX complex also participates in the miRNA transport [140]. 

The primary function of miRNA is to regulate post-transcriptional gene modification of the target genes through two major operational pieces of machinery: (1) transcript cleavage, and (2) translation repression [103,141,142]. The conventional, general rule that dictates the mode of action of the small RNAs is the level of sequence complementarity between them and their corresponding targets because the small RNAs, which are associated with transcript cleavage, require maximum sequence complementarity [143]. There was a tremendous misconception regarding the mode of action of the plant miRNAs, which happened due to the impeccable sequence complementarity between the plant miRNAs and their corresponding mRNAs [141,142]. It is true that a good amount of sequence complementarity is favorable for RNA cleavage, but it is not that stubborn toward translational repression. It has been confirmed experimentally by many research groups that a high degree of sequence complementarity is present between the miRNAs and their targets to finally bring about miRNA-mediated inhibition of translation [144,145,146]. Citable examples of target genes and their corresponding miRNAs (which downregulate the degree of protein translation) are *APETALA2 (AP2)*/miR172, *SQUAMOSA PROMOTER BINDING PROTEIN-LIKE 3 (SPL3)*/miR156, *COPPER/ZINC SUPEROXIDE DISMUTASE 2* (Cu/ZnSOD)/miR398, and *SCARECROW-LIKE PROTEIN 4 (SCL4)*/miR171 [144,147,148,149]. 

The above discussion validates the point that sequence complementarity is not a mandatory criterion for the mode of action of miRNAs in plants. Recent research has also established that the miRNA targets (mRNAs) are stuck on the ribosomes in the endoplasmic reticulum (ER) [150,151,152], which implies that translational inhibition might be a major instrument that is operational on a larger percentage of miRNA targets than what is expected. Because plant miRNAs show a significant level of sequence complementarity with their corresponding target mRNAs, the use of bioinformatics tools has proved to be extremely useful in predicting and subsequently validating the miRNA targets in plants [153]. As stated above, with regard to the three types of operational machinery used by miRNA to perform its function, here we discuss both of them chronologically.

#### 3.1.1. Transcript Cleavage 

The phenomenon of miRNA-directed RNA cleavage is popularly named slicing. This phenomenon occurs at a predetermined location in the targeted mRNA [154]. RNAs were identified throughout the genome with a 5′ monophosphate. It was observed that quite a large number of miRNA targets face transcript cleavage [155]. Transcript cleavage is achieved by the PIWI domain of the AGO proteins, which orients itself similar to the folding pattern of RNase-H and possesses endonuclease activity. The AGO1 protein, which acts as the primary effector in *Arabidopsis thaliana*, undergoes cleavage in the above-stated manner, and this activity also occurs with a plethora of other effectors such as AGO2, AGO4, AGO7, and AGO10 [156,157,158,159,160,161]. In Arabidopsis, the 5′-to-3′ exonuclease is designated as EXORIBONUCLEASE 4 (XRN4), and it degrades the 3′ fragments [162]. The same happens with the 5′ cleavage fragments, which are almost undetectable in the wild-type species because of their speedy degradation. *Chlamydomonas reinhardtii* shows an interesting phenomenon in which the nucleotidyl transferase MUT68 first polyadenylates the 5′ fragments, and then degradation takes place via the cytoplasmic exosome [163]. The *Arabidopsis* homolog of MUT68, called HEN SUPPRESSOR 1 (HESO1), along with its paralogue UTP: RNA URIDYLYLTRANSFERASE 1(URT1) carry out polyuridylation of the 5′ fragments both in vitro and in vivo [164,165], and RISC-INTERACTING CLEARING 3′-5′ EXORIBONUCLEASE 1(RICE1) degrades the uridylated 5′ pieces. If these fragments are left undegraded, then they might stock-pile in plants and result in the formation of a catalytically inactive variety of RICE1 [166]. The potential role played by cytoplasmic exosomes is also significant because the subunits {SUPERKILLER 2, (SKI2), SKI3, SKI8,} of the cofactor are essential for the breakdown of the RISC-bread 5′ shreds [167].

#### 3.1.2. Translation Inhibition

The phenomenon of translation inhibition is not as common in plants as is transcript cleavage because miRNA-guided cleavage is active all over; in addition, there are bottlenecks in estimating the protein levels because of the lack of worthwhile antibodies. Some examples can be cited that show miRNA-induced translation inhibition, and those are in plants in which *AP2* is regulated by miR172, and *SPL3* is regulated by miR156/7 [147,148,149]. The necessary components required to carry out miRNA-mediated translation inhibition involve the microtubule-serving enzyme KATANIN1 (KTN1) [144]; the processing body (P body) component VARICOSE (VCS) [144]; SUO protein, which is a GW repeat [145]; and the ALTERED MERISTEM PROGRAM1 (AMP1), which is the ER membrane protein [146]. It had been established that mutation in any of these above-stated genes could significantly hinder the process of miRNA-guided translation repression, and these events vividly indicate that transcript cleavage and translation inhibition are two independent lines of action with no link with each other. Genome-wide RNA profiling with 5′ monophosphates in *Arabidopsis thaliana* is performed to obtain an idea of the RNA degradation phenomenon, which clearly indicates that co-translational mRNA breakdown takes place for the majority of genes, which involves a plethora of miRNA targets [150,152]. A notable inference has been drawn from the entire study, i.e., even if the mode of action of the miRNAs is “RNA cleavage,” then also the potential targets are the translating miRNAs. Although the entire molecular mechanism of translation inhibition is yet unclear, in vitro studies hint that plant miRNA has the capacity to hinder the activity and operations of the ribosomes [168]. However, an immeasurable amount of information and knowledge has been gained about all the lead role players participating in miRNA biogenesis, mode of action etc. Nonetheless, uncertainty still persists about the subcellular locations where these activities are carried out. Activities such as the process of formation of D-bodies containing the dicing complexes are yet unknown. Questions such as the method of association of AGO1 with the Endoplasmic Reticulum (ER) and membrane-bound polysomes or how membrane-bound polysome affects miRNA-guided phasiRNA biogenesis are still in a haze. 

Since AGO1 has a dual behavior of associating not only with miRNAs but also with siRNAs from endogenous sequences such as transposons, phasiRNA loci, and exogenous sequences such as viruses, transgenes, etc. The subcellular division and distribution of AGO1 in the middle of the cytosol, endomembrane compartments, the nucleus, and the cytoplasm have a significant impact on the scheme of action of various small RNAs. The lack of a complete and clear understanding and updated knowledge about the subcellular locations of miRNA biogenesis, and activity, deprive us of a vivid picture of miRNAs and their crosstalk with siRNAs [169].

### 3.2. microRNA Mediated Silencing in Plants

RNAi technology, especially the small non-coding RNAs involving miRNA, has been proven to possess immense potential to act as an attractive tool for researchers worldwide in the design and generation of plants with refined and upgraded traits by tailoring both advantageous and disadvantageous genes. The functions of individual genes can be comprehended with this powerful tool and have proven to be of immense utility to molecular crop breeders to generate improved varieties. Non-coding small RNAs have been widely used in silencing genes of interest by the RNAi technique. Adopting the method of overexpression of miRNA is especially prevalent, along with the process of introducing artificially synthesized miRNA targeting the gene of interest. 

Small non-coding RNAs have been of immense use in the field of agricultural biotechnology, and their mass utilization has led to food safety because the crop cultivars thus generated have improved agronomic traits such as higher yield and nutritional value. Interactions of miRNAs with their targets is an interesting domain of research, and it has thrown light on the procedures of post-transcriptional gene regulation and a labyrinth of signaling cascades that have complete control of the plant stress responses. Though there are immense benefits of small non-coding RNAs in improving agriculture, there are certain drawbacks as well. Modification of the expression of a certain gene or its corresponding miRNA might rebound, and certain pleiotropic changes might crop up in the plant’s morphology and its development. Therefore, a complete understanding of the mechanism of miRNAs is mandatory before designing transgenic strategies. 

miRNA strategies are somewhat confusing when implemented into application. The strategy which has proved fruitful for a certain plant species might not achieve success for another species, and this phenomenon is attributed to the various types of regulation of the evolutionarily conserved miRNA across different species [170]. Synthetically prepared miRNAs are of immense use in the market because they can overcome the potential drawbacks and limitations posed by siRNAs given that they bring about gene silencing with the utmost precision and finesse. The chances of off-target effects are significantly lesser with miRNA-based-RNAi compared to siRNAs because they require lesser nucleotides (single 21/22 nt sequence) to identify the target sequence. This trait of miRNAs makes miRNA-based RNAi an extremely popular one because it becomes a cakewalk to target one specific gene in a family of closely related genes. Genetically engineered crops which contain constructs that encode for a heterologous protein or overexpress a certain protein are constitutionally dissimilar from the transgenics which are prepared by RNAi technology because the gene suppression cassettes express non-coding RNAs only. Hence, it can be inferred that the transgenic crops with traits introduced by RNA-based methods are secure for human and animal consumption and do not demand intense research on the digestibility issues of the newly launched RNA component inside the cell. A point of concern regarding the biosafety of the RNAi transgenics is that gene silencing by chromatin alteration and recast might have adverse effects, which could be passed on through progenies. Although these hindrances and limitations regarding the mindset of scientists are relatively small in number, it has been proven successfully that strategies executed based on small non-coding RNAs have immense and immeasurable potential to bring about a tremendous surge in crop productivity and a significant soar in their nutritional value.

### 3.3. Tweaking the miRNAs to Improve Desirable Traits in Plants

The miRNAs play a vital role in plant growth and development during stress resistance, whether biotic or abiotic (Table 4). These categories of RNAs are established regulators of various complex networks. Several research and review articles have vouched for the role of miRNAs in plant growth and development [171,172,173]. In unraveling the complex regulatory mechanisms that control numerous developmental stages in a plant, a plethora of miRNAs and their complex genetic networks came into the spotlight [172]. Different research groups have identified miRNA target modules, such as miR156-*SQUMOSA PROMOTER BINDING PROTEIN-LIKE (SPL)*, miR172-*APETALA2 (AP2),* and miR159-*MYELOBLASTOSIS (MYB),* which regulate the various key transition phases during plant development [172,174]. To cite an example, in the model plant Arabidopsis, the miR156-*SPL* module is a potential negative regulator during the germination–vegetative growth–reproductive phase transition. A drop in the level of miR156 prompts *SPL* expression, which subsequently accelerates changes, whereas miR172-*AP2* works precisely the opposite way [175]. miRNAs multitask, as they act as coordinators and connect the regulatory networks with various phytohormones such as gibberellic acid (GA) and abscisic acid (ABA), thus exercising a tight grip over germination and dormancy in plants [176,177,178]. Extensive global research has clearly established that miRNA acts as an efficient mediator in numerous signaling pathways governing plant development. Apart from these, miRNAs are adept at performing via an integrative mode controlling a solo function [173]. It has also been established that the various existing isoforms of a single miRNA family can play an active part in regulating different physiological functions via the same or various other genes [179]. 

The literature demonstrates that miRNAs regulate gene expression in dealing with various stress responses, both biotic and abiotic. Their versatile role has been demonstrated in the model plant Arabidopsis and other cereal crops such as rice, wheat, maize, and barley [175]. In many plants, it has been clearly witnessed that a wide range of miRNA expression exists to fight out various stress factors. However, the anomaly observed is that only a handful of miRNA-target modules can regulate the expression pattern of the target genes responsible for the specific stress response. It has also been observed that this pattern is conserved throughout various species of plants [180]. The role of these conserved miRNA-target modules is also to confer stress resistance to plants by participating in different metabolic pathways. Research has proved that numerous well-established miRNA-target modules, a citable example being mi-R398-*COPPER/ZINC SUPEROXIDE DISMUTASE (CSD)*, can mitigate the detrimental effects of various stress factors. miRNAs interact with transcription factors (TFs) to regulate the signaling of stress-related hormones such as Auxin, Ethylene, ABA, and GA during drought conditions [181]. In a similar case, it is seen that the miR169-*NFY* module regulates plant stress response during water stress. Expression of *NF-Y* is increased in stomatal guard cells to control their aperture, which consequently facilitates drought tolerance [182]. In Arabidopsis, the enhanced heat tolerance is attributed to the increased expression of miR398, which downregulates the specific targets (*CSD1, CSD2*, and *COP-PER CHAPERONE OF CSD (CCD)* [183]. The highly conserved miR394-*LCR* module is an active participant in cold-stress response in plants, and overexpressed miR394a Arabidopsis plants exhibit cold tolerance because they downregulate the *LCR* gene [184]. In bentgrass (*Agrostis stolonifera*), tolerance towards saline stress is conferred by the overexpression of the osa-miR319a module [185]. 

The miRNA-targeted modules regulate tolerance towards biotic stress factors such as an attack of bacteria, fungi, virus, and various pests [180]. In Arabidopsis, miR393-*TRANSPORT INHIBITOR RESPONSE1 (TIR1)*, *AUXIN SIGNALING F-BOX1 (AFB2)*, and *AFB3* are the modules responsible for the defense response against *Pseudomonas syringae* PV. tomato DC3000. The authors of one study [186] also worked on the miR773-*METHYLTRANSFERASE2 (MET2)* module, for which enhanced resistance was observed as a part of pathogen-associated triggered immunity in the case of fungus. In the case of viral defense, a typical example is rice, in which the miR528-*ASCORBATE OXIDASE (AO)* module facilitates the aggregation of reactive oxygen species (ROS). When the rice stripe virus (RSV) strikes, miR528 covered by AGO 18 comes into action, leading to the rise in ROS levels which render antiviral defense.

**Table 4:** Genetic alteration of miRNAs in plants to improve the desirable traits.

Plant	miRNA	Targeted Towards	Traits Involved	References
Tomato	miR482e-3p	NBS-LRR class proteins	Improved fungal resistance	[187]
Tomato	miR482/2118	Leucine-rich repeat protein	Improved bacterial resistance	[188]
Arabidopsis	miR827	Nitrogen limitation adaptation	Improved nematode resistance	[189]
Cotton	miR166b	Mitochondrial ATP synthase of Bemisia tabaci	Improved insect resistance	[190]
Arabidopsis	miR773	Methyltransferase 2	Improved fungal resistance	[186]
Arabidopsis	miR408	uclacyanin	Improved biomass grain yield	[191]
Rice	miR396	GRF6	Yield increase	[192]
Arabidopsis	miR319	TCP transcription factors	Delayed flowering	[193]
*Camelina sativa*	miR159	Fatty acyl-ACP thioesterases	Improved seed quality	[194]
*Salvia miltiorrhiza*	miR160	ARF10, 16, 17	Improved biomass	[195]
Tomato	miR858	SlMYB7-like	Increased anthocyanin	[196]
Arabidopsis	miR398	CSD1 and CSD2	Improved resistance against salt and heavy metal	[197]
Wheat	miR408	Phosphate transporter	Improved biomass and Pi acquisition	[198]
Rice, tobacco	miR444	OsMADS23,27a,27b,57 and Tobacco genes (NRTs/AEEs)	Improved N and Pi acquisition	[199,200]
Rice	miR528	L-ascorbate oxidase	Improved resistance against RBSDV	[201]
Rice	miR444	MIKC^C^-type MADS-box proteins	Improved resistance against RSV	[202]
Arabidopsis	miR156	SPL9	Improved resistance against insect	[203]
Rice	miR396	GRF8	Improved resistance against insect and fungi	[204,205]
Arabidopsis	miR159	sex lethal (Sxl) protein, acetylcholinesterase (AChE) and orcokinin (Orc)	Improved resistance against insect	[206]
Tomato, alfalfa	miR156	SPL	Improved tolerance against drought	[207,208]
Arabidopsis	miR402	DEMETER-LIKE Protein 3	Improved tolerance against salinity	[209]
Cotton	miR160	ARF10, ARF16, ARF17	Improved tolerance against Heat	[210]
Sunflower	miR396	HaWRKY6	Improved tolerance against heat	[211]
Rice	miR166	HD-Zip family proteins	Improved tolerance against Cd	[212]
Arabidopsis	miR402	DEMETER-LIKE Protein 3	Improved tolerance against salinity and increased seed germination	[209]
Tomato	miR169	SlNF-YA1/2/3, SlMRP1	Improved resistance against drought	[170]
Wheat	miR408	NAC domain protein and protein phosphatase	Increased uptake of Potassium	[213]

## 4. Trans-Acting siRNA

Gene silencing in eukaryotes was observed many years ago and has been a fascinating topic for researchers, but the mechanism behind this phenomenon was quite unclear then. Hence, this process of silencing (s-RNA) was called and identified by various names, viz., RNA interference, co-suppression, quelling, etc. are among the plethora of short sRNAs identified, mainly miRNA and siRNA rampant in plants. siRNA has been categorized into various types and among them, one is trans-acting siRNA (tasiRNA), which is being studied in great detail. tasiRNAs originate from non-coding RNA (ncRNA) precursors, and the most discerning feature of tasiRNA biogenesis lies in the need for a miRNA-dependent pathway for the production of a single-stranded RNA (ssRNA) precursor [214,215]. *Arabidopsis thaliana*, the universal model plant, first gave substantial evidence of the miRNA-activated secondary siRNA generating loci. In this case, the non-coding RNA molecules rampantly produced siRNAs that repressed the gene expression of all those genes which were unassociated with their precursor molecules; hence, the nomenclature of *trans*-acting siRNAs developed (tasiRNAs). *Arabidopsis thaliana* has four families of tasiRNA-generating loci (*TAS1-4*). miR173 targets *TAS1* and *TAS2,* whereas the biogenesis of TAS3 and TAS4 happens in the presence of miR390 and miR828, respectively [216,217,218]. Extra *TAS* genes such as *TAS5-10* have been speculated to exist in other plants as well; hence, global research focusing on secondary siRNAs is being performed by various research groups [219,220,221,222]. 

### 4.1. Biogenesis and Structure

**Figure 4:**
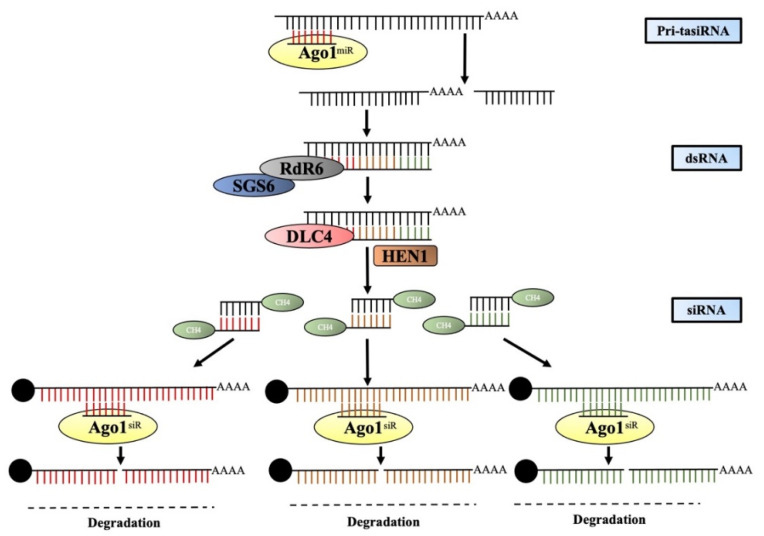
The genomic *TAS* locus produces the pri-tasi transcripts. They are cut by miRNA by AGO1 and transformed into a double-strand by SGS3 and RDR6 jointly. Moreover, 21-nt-diced siRNA is produced and is methylated by HEN1. Finally, the target transcript is degraded by the 21-nt siRNA in a sequence-specific manner.

tasiRNAs are 21-nt structures that are generated from non-protein-coding primary TAS transcripts. These primary *TAS* transcripts are capped and polyadenylated as per the general rule, and it has been observed that the generation of tasiRNAs is set off by the breakup of these primary *TAS* transcripts (pri-TASs) by the specific mRNAs, and these transcripts possess a binding site predominantly for 22-nt miRNA. tasiRNAs are proactive in the methylation process of the TAS DNA, but they do not play any role in the generation of the TAS transcripts [223]. The cleaved product formed by the miRNAs is brought into a steady state by the suppressor of gene silencing 3 (SGS3) [12] and is transformed into a dsRNA by RDR6. This transitional state is then acted upon by DCL4 and dsRNA binding protein 4 (DRB4) to form 21 nt siRNAs. These intermediatory siRNAs are integrated into the AGO-RISC complex, the aim of which is to target complementary sequences [12,215,216]. Research and observation have led to the inference that AGO1 and AGO7 proteins are linked with the activities of tasiRNAs. Irrespective of the type of small non-coding RNA, 3′-end methylation of all is carried out by HUA ENHANCER 1 (HEN1) (Figure 4). 

In the universal model plant *Arabidopsis thaliana*, there are eight tasiRNA-generating loci, and all of them have been classified into four TAS groups (TAS1-TAS4). Two 22 nt complexes, namely miR173::AGO1 RISC and miRNA::AGO1 RISC, start the generation of tasiRNA from TAS1a, b, c, TAS2 RNAs, and TAS4, respectively [157,224,225]. A dazzling exception exists in this entire scenario of tasiRNA generation, which concerns TAS3 because it is from that group that the generation of tasiRNA commences by miR390:AGO7 RISCs, each of which are 21 nt long.

Here too lies a hindrance similar to that of all reactions. Only the 3′ closest site of TAS3 could be cut, but the 5′ end fails to cleave the target because a high degree of sequence mismatch with miR390 has been found [157]. The most interesting fact that has been noticed by scientists is that the 3′ miR390 target site is not a mandatory part of the biogenesis process of tasiRNAs. In place of tasiRNAs, phasiRNAs can be generated. The miR390 can be replaced by many other miRNAs provided that they continue the cleavage [157]. Secondary siRNA generation is totally stopped on certain occasions, such as when any type of undesired change takes place in the miR390-binding site. Thus, the TAS3 5′ region is to be protected at any cost because it triggers the production of tasiRNAs on a large scale. Apart from these four families in the model plant *Arabidopsis thaliana*, many other plant sources that generate tasiRNAs similarly have been detected. Some solid examples are the following: TAS5 was first identified and reported in tomatoes, three TAS6 genes have been reported in moss, and a long list of TAS genes (TAS7-9) have been reported in grapevine (Sanan-Mishra et al. 2021) [226]. Much research is being conducted worldwide, yet the tasiRNAs belonging to TAS7-10 genes are yet to be fully characterized. The most interesting fact is that a plethora of non-coding RNAs generates tasiRNAs when given sufficient trigger by the initiator miRNAs [226]. 

### 4.2. tasiRNA Mediated Silencing in Plants

The function of the tasiRNAs is quite unique. The target site of the TASI tasiRNAs is the pentatricopeptide repeat which comprises the pentatricopeptide-repeat protein (PPR) genes and one or two other genes as well [216]. On the other hand, the TAS2-tasiRNA attacks solely the PPR genes. Findings reveal that *Arabidopsis* contains a count of almost 500 PPR genes, the majority of which are associated with the abiotic stress response, out of which only a selected few are the potential targets of TASI tasiRNAs. TASI controls and fine-tunes several heat stress-related transcription factors [227]. In *Arabidopsis thaliana*, an elevated thermotolerance level is achieved by the increased magnitude of “heat-induced TASI target 1 and 2 (HTT1, HTT2)” due to the reduced quantity of TASI-tasiRNA on being subjected to high heat. Kume et al. 2010 identified the reason behind chilling tolerance. He obtained a clear picture in which TASI-derived tasiRNAs gathered in significantly lesser amounts at 4 °C, so as a consequence of such a trigger, the gene expression of target genes such as *At151670*, *At4g29760,* and *At5g18040* peaked in response in order to survive the severe cold [228]. TAS3-tasiRNAs are the conserved group of tasiRNAs, and they target the auxin response factors (ARF), thus guaranteeing a tight grip over a wide range of biological activities. In *Arabidopsis thaliana*, TAS3 can self-propagate and give rise to a minimum number of nine tasiRNAs. Out of these nine, two generally aim for two ARFs such as ARF3 and ARF4, whereas the remaining seven might target other ARFs. The association of ARF3 with tasiRNA predominantly determines the nature of the adaxial surface of the leaves. 

Root architecture, leaf morphology, flower architecture, stress responses, hormonal crosstalk, developmental transition, embryo development, and other characteristics are controlled by the tasiRNA–ARF complex. It was noted that defective biogenesis of TAS3-tasiRNA resulted in unnatural floral development such that the development of juvenile to fully mature flowers was too fast [229]. This tasiRNA–ARF complex is also in charge in various other plants (*Medicago tranculata*, *Lotus japonicus*, *Zea mays*, *Pyrus serotina,* etc.) [136]. This module plays a role in auxin signaling and nitrogen sensitivity in the moss *Physcomitrella patens*, which clearly indicates that this module was coopted in the evolutionary process of lower plants [230]. tasiRNAs are equally responsible for AP2 transcription factors that are present in bryophytes. 

Around 5047 tasiRNAs are found to be active in *Arabidopsis thaliana* and to downregulate the *myb* genes, just about nine tasiRNAs are begotten by TAS4 when activated by the 22-nt miR828. The most prepotent tasiRNAs {TAS4-tasiRNA81(-)} cut MYB-90 (PAP2), MYB-75 (PAP1), and MYB-113 [218], all of which are associated with the anthocyanin buildup pathway and the origin of trichomes in *Arabidopsis* leaves. Eventually, these tasiRNAs are negative regulators of trichome initiation [231]. miR828 actively regulates MYB2D/MYB2A genes in cotton and, subsequently, these genes give rise to tasiRNAs which also hinder the formation of cotton fiber. The miR828–TAS4 complex is also present in numerous dicot plants as per reports (not reported in monocots), and participation of miR828-TAS4 has been observed in apples as well [232]. 

### 4.3. Tweaking the tasiRNAs to Improve the Desirable Traits in Plants

TasiRNAs, a subcategory of siRNA (broad category sncRNAs), have a prominent role in plant vegetative growth and development [233]. The miR390-AGO7 complex gives rise to the phased *TAS3*-tasiRNAs, which target the *ARF* gene family and govern numerous biological processes in the plant. *TAS3*-tasiRNAs form a regulatory complex with *ARF* (*TAS3*-tasiRNAs–ARF), and this is considered to be the most conserved complex which plays a crucial role in the regulation of biological processes such as developmental transitions, root structure, embryo development, shoot apical meristem development, root structure, and leaf morphology along with flower and phytohormone crosstalk [173]. In the model plant Arabidopsis, miR828 targets the *MYB* genes (*PRODUCTION OF ANTHOCYANIN PIGMENT1*, *PAP1*, *PAP2*, and *MYB113*), which in turn triggers the *TAS4*-tasiRNAs and facilitates the anthocyanin biosynthetic pathway [234]. 

TasiRNAs have also been reported to be actively involved in regulating the plant stress response. A classic example of thermotolerance was reported in the model plant Arabidopsis, where miRNA173 is formed by the participation of numerous factors such as *HEAT-INDUCED TAS1 TARGET1 (HTT1)*, *HTT2* (both are targets of TAS1 (trans-acting siRNA precursor 1)-derived tasiRNAs) and the plants that had been overexpressed with genes *HTT1* and *HTT2* showed increased resistance towards thermal stress. In contrast, plants with high TASI-siRNAs and low *HTT* gene expression levels displayed susceptibility to heat stress [227]. Similarly, to combat biotic stress overexpressing two tasiRNAs originating from TAS1 and TAS2 loci rendered resistance to plants against the deadly fungus *Botrytis cinerea* [235]. 

## 5. Present Challenges Regarding Non-Coding RNAs

Traditional methods for the identification and prediction of ncRNAs include microarray, RNA sequencing, or fluorescence in situ hybridization (FISH) although paired-end strand-specific RNA sequencing may provide better transcript information in the near future. New and improved prediction tools for the tissue-specificity and chromosome location of ncRNAs are required on an urgent basis. For instance, to better predict the chromosomal location of ncRNAs, single-molecule RNA FISH (smFISH) was invented to aid the visualization of individual RNAs using target specific fluorescently labelled oligonucleotide probes [236]. With the increasing advancement of tools and technologies, methods of mapping the knowledge gained from ncRNAs in the animal system to the plant system could be possible. Apart from the small non-coding RNAs in plants, deciphering of the detailed mechanism of biogenesis and mode of action of long non-coding RNAs (lncRNA), which is still in its early phase, needs to be performed. With the advent of new technologies such as CRISPR/Cas and large-scale RNA profiling, more and more new classes of ncRNAs are coming to light, such as the discovery of a novel small nucleolar RNA (snoRNA) or tRNA-ended lncRNAs, which are not yet found in plants [237]. Moreover, to efficiently implement the existing knowledge of ncRNAs for creating improved engineered variety of crops, it is a matter of priority to develop a trait-specific candidate ncRNA catalogue.

## 6. Concluding Remarks and Future Prospects

miRNAs have long been associated with the cellular regulatory network, but with the emergence of new research, other non-coding RNAs such as siRNAs, tasiRNAs, or long non-coding RNAs have also been proven to be significant regulatory molecules. Over the years, comprehensive research on the biogenesis and mode of action of siRNAs/miRNAs proved valuable because that information is rapidly translated into plant–pathogen interaction studies to develop biotic stress-resilient crops. Some newly discovered non-coding RNAs, such as tasiRNAs, and phasiRNAs are still in their infancy and need thorough research to decipher how they are actively involved in the plant’s overall development. With the increasing population crisis, disease-resistant smart crops with enhanced productivity would be a top priority to feed the teeming millions. Exploiting non-coding RNAs might be the key to bringing out the solution because they are involved both in the developmental and immunity aspects of a plant’s life cycle.

## Data Availability

Not applicable.

## References

[1] Pauli A., Rinn J.L., Schier A.F. (2011). Non Coding RNAs Regulation in Embryogenesis. Nat. Rev. Genet..

[2] Rai M.I., Alam M., Lightfoot D.A., Gurha P., Afzal A.J. (2019). Classification and Experimental Identification of Plant Long Non-Coding RNAs. Genomics.

[3] Ariel F., Romero-Barrios N., Jégu T., Benhamed M., Crespi M. (2015). Battles and Hijacks: Noncoding Transcription in Plants. Trends Plant Sci..

[4] Cech T.R., Steitz J.A. (2014). The Noncoding RNA Revolution—Trashing Old Rules to Forge New Ones. Cell.

[5] Peschansky V.J., Wahlestedt C.W.C. (2014). Non-Coding RNAs as Direct and Indirect Modulators of Epigenetic Regulation. Epigenetics.

[6] Ponjavic J., Ponting C.P., Lunter G. (2007). Functionality or Transcriptional Noise? Evidence for Selection within Long Noncoding RNAs. Genome Res..

[7] Brosnan C.A., Voinnet O. (2009). The Long and the Short of Noncoding RNAs. Curr. Opin. Cell Biol..

[8] D’Ario M., Griffiths-Jones S., Kim M. (2017). Small RNAs: Big Impact on Plant Development. Trends Plant Sci..

[9] Campalans A., Kondorosi A., Crespi M. (2004). Enod40, a Short Open Reading Frame-Containing MRNA, Induces Cytoplasmic Localization of a Nuclear RNA Binding Protein in Medicago Truncatula. Plant Cell.

[10] Guleria P., Mahajan M., Bhardwaj J., Yadav S.K. (2011). Plant Small RNAs: Biogenesis, Mode of Action and Their Roles in Abiotic Stresses. Genom. Proteom. Bioinform..

[11] Zhang L., Xiang Y., Chen S., Shi M., Jiang X., He Z., Gao S. (2022). Mechanisms of MicroRNA Biogenesis and Stability Control in Plants. Front. Plant Sci..

[12] Yoshikawa M., Peragine A., Mee Y.P., Poethig R.S. (2005). A Pathway for the Biogenesis of Trans-Acting SiRNAs in Arabidopsis. Genes Dev..

[13] Liu Y., Teng C., Xia R., Meyers B.C. (2020). PhasiRNAs in Plants: Their Biogenesis, Genic Sources, and Roles in Stress Responses, Development, and Reproduction. Plant Cell.

[14] Napoli C., Lemieux C., Jorgensen R. (1990). Introduction of a Chimeric Chalcone Synthase Gene into Petunia Results in Reversible Co-Suppression of Homologous Genes in Trans. Plant Cell.

[15] Romano N., Macino G. (1992). Quelling: Transient Inactivation of Gene Expression in Neurospora Crassa by Transformation with Homologous Sequences. Mol. Microbiol..

[16] Guo S., Kemphues K.J. (1995). Par-1, a Gene Required for Establishing Polarity in C. Elegans Embryos, Encodes a Putative Ser/Thr Kinase That Is Asymmetrically Distributed. Cell.

[17] Fire A., Xu S., Montgomery M.K., Kostas S.A., Driver S.E., Mello C.C. (1998). Potent and specific genetic interference by double-stranded RNA in Caenorhabditis elegans. Nature.

[18] Fire A. (1999). RNA-Triggered Gene Silencing. Trends Genet..

[19] Carthew R.W. (2001). Gene Silencing by Double-Stranded RNA. Curr. Opin. Cell Biol..

[20] Dalmay T., Hamilton A., Rudd S., Angell S., Baulcombe D.C. (2000). An RNA-Dependent RNA Polymerase Gene in Arabidopsis Is Required for Posttranscriptional Gene Silencing Mediated by a Transgene but Not by a Virus. Cell.

[21] Smardon A., Spoerke J.M., Stacey S.C., Klein M.E., MacKin N., Maine E.M. (2000). EGO-1 Is Related to RNA-Directed RNA Polymerase and Functions in Germ-Line Development and RNA Interference in C. Elegans. Curr. Biol..

[22] Bernstein E., Caudy A.A., Hammond S.M., Hannon G.J. (2001). Role for a bidentate ribonuclease in the initiation step of RNA interference. Nature.

[23] Hamilton A.J., Baulcombe D.C. (1999). A Species of Small Antisense RNA in Posttranscriptional Gene Silencing in Plants. Science.

[24] Zamore P.D., Tuschl T., Sharp P.A., Bartel D.P. (2000). RNAi: Double-Stranded RNA Directs the ATP-Dependent Cleavage of MRNA at 21 to 23 Nucleotide Intervals. Cell.

[25] Elbashir S.M., Lendeckel W., Tuschl T. (2001). RNA Interference Is Mediated by 21- and 22-Nucleotide RNAs. Genes Dev..

[26] Hammond S.M., Boettcher S., Caudy A.A., Kobayashi R., Hannon G.J. (2001). Argonaute2, a Link between Genetic and Biochemical Analyses of RNAi. Science.

[27] Matranga C., Tomari Y., Shin C., Bartel D.P., Zamore P.D. (2005). Passenger-Strand Cleavage Facilitates Assembly of SiRNA into Ago2-Containing RNAi Enzyme Complexes. Cell.

[28] Rand T.A., Petersen S., Du F., Wang X. (2005). Argonaute2 Cleaves the Anti-Guide Strand of SiRNA during RISC Activation. Cell.

[29] Tomari Y., Zamore P.D. (2005). Perspective: Machines for RNAi. Genes Dev..

[30] Elbashir S.M., Harborth J., Lendeckel W., Yalcin A., Weber K., Tuschl T. (2001). Duplexes of 21±nucleotide RNAs Mediate RNA Interference in Cultured Mammalian Cells Sayda. Nature.

[31] Elbashir S.M., Martinez J., Patkaniowska A., Lendeckel W., Tuschl T. (2001). Functional Anatomy of SiRNAs for Mediating Efficient RNAi in Drosophila Melanogaster Embryo Lysate. EMBO J..

[32] Mourrain P., Béclin C., Elmayan T., Feuerbach F., Godon C., Morel J.B., Jouette D., Lacombe A.M., Nikic S., Picault N. (2000). Arabidopsis SGS2 and SGS3 Genes Are Required for Posttranscriptional Gene Silencing and Natural Virus Resistance. Cell.

[33] Cogoni C., Macino G. (1999). Gene Silencing in Neurospora Crassa Requires a Protein Homologous to RNA-Dependent RNA Polymerase. Nature.

[34] Brown R.D., Mattoccia E., Tocchini-Valentini G.P. (1972). On the Role of RNA in Gene Amplification. Acta Endocrinol. Suppl..

[35] Mello C.C., Conte J.D. (2004). Revealing the World of RNA Interference. Nature.

[36] Pratt A.J., MacRae I.J. (2009). The RNA-Induced Silencing Complex: A Versatile Gene-Silencing Machine. J. Biol. Chem..

[37] Peters L., Meister G. (2007). Argonaute Proteins: Mediators of RNA Silencing. Mol. Cell.

[38] Meister G. (2013). Argonaute Proteins: Functional Insights and Emerging Roles. Nat. Rev. Genet.

[39] Nakanishi K., Weinberg D.E., Bartel D.P., Patel D.J. (2012). Structure of Yeast Argonaute with Guide RNA. Nature.

[40] Elkayam E., Kuhn C.D., Tocilj A., Haase A.D., Greene E.M., Hannon G.J., Joshua-Tor L. (2012). The Structure of Human Argonaute-2 in Complex with MiR-20a. Cell.

[41] Wang Y., Sheng G., Juranek S., Tuschl T., Patel D.J. (2008). Structure of the Guide-Strand-Containing Argonaute Silencing Complex. Nature.

[42] Parker J.S., Roe S.M., Barford D. (2005). Structural Insights into MRNA Recognition from a PIWI Domain-SiRNA Guide Complex. Nature.

[43] Iwasaki S., Kobayashi M., Yoda M., Sakaguchi Y., Katsuma S., Suzuki T., Tomari Y. (2010). Hsc70/Hsp90 Chaperone Machinery Mediates ATP-Dependent RISC Loading of Small RNA Duplexes. Mol. Cell.

[44] Fomenko A.I., Donchenko G.V., Stepanenko S.P. (1996). Effect of Withdrawal of Phenazepam and Nicotinamide on the State of the Systems of Reception of Benzodiazepines and NAD. Ukr Biokhim Zh.

[45] Khvorova A., Reynolds A., Jayasena S.D. (2003). Functional SiRNAs and MiRNAs Exhibit Strand Bias. Cell.

[46] Wang Y., Juranek S., Li H., Sheng G., Wardle G.S., Tuschl T., Patel D.J. (2009). Nucleation, Propagation and Cleavage of Target RNAs in Ago Silencing Complexes. Nature.

[47] Miyoshi K., Tsukumo H., Nagami T., Siomi H., Siomi M.C. (2005). Slicer Function of Drosophila Argonautes and Its Involvement in RISC Formation. Genes Dev..

[48] Djuranovic S., Nahvi A., Green R. (2012). MiRNA-Mediated Gene Silencing by Translational Repression Followed by MRNA Deadenylation and Decay. Science.

[49] Iwakawa H.O., Tomari Y. (2022). Life of RISC: Formation, Action, and Degradation of RNA-Induced Silencing Complex. Mol. Cell.

[50] Winter J., Diederichs S. (2011). Argonaute Proteins Regulate MicroRNA Stability: Increased MicroRNA Abundance by Argonaute Proteins Is Due to MicroRNA Stabilization. RNA Biol..

[51] Vaucheret H., Vazquez F., Crété P., Bartel D.P. (2004). The Action of ARGONAUTE1 in the MiRNA Pathway and Its Regulation by the MiRNA Pathway Are Crucial for Plant Development. Genes Dev..

[52] Derrien B., Baumberger N., Schepetilnikov M., Viotti C., De Cillia J., Ziegler-Graff V., Isono E., Schumacher K., Genschik P. (2012). Degradation of the Antiviral Component ARGONAUTE1 by the Autophagy Pathway. Proc. Natl. Acad. Sci. USA.

[53] Kobayashi H., Shoji K., Kiyokawa K., Negishi L., Tomari Y. (2019). VCP Machinery Mediates Autophagic Degradation of Empty Argonaute. Cell Rep..

[54] Shi C.Y., Kingston E.R., Kleaveland B., Lin D.H., Stubna M.W., Bartel D.P. (2020). The ZSWIM8 Ubiquitin Ligase Mediates Target-Directed MicroRNA Degradation. Science.

[55] Han J., Lavigne C.A., Jones B.T., Zhang H., Gillett F., Mendell J.T. (2020). A Ubiquitin Ligase Mediates Target-Directed MicroRNA Decay Independently of Tailing and Trimming. Science.

[56] Rajam M.V. (2020). RNA Silencing Technology: A Boon for Crop Improvement. J. Biosci..

[57] Ghag S.B. (2017). Host Induced Gene Silencing, an Emerging Science to Engineer Crop Resistance against Harmful Plant Pathogens. Physiol. Mol. Plant Pathol..

[58] Shin Y.H., Lee S.H., Park Y.D. (2020). Development of Mite (Tetranychus Urticae)-Resistant Transgenic Chinese Cabbage Using Plant-Mediated RNA Interference. Hortic. Environ. Biotechnol..

[59] Hussain T., Aksoy E., Çalışkan M.E., Bakhsh A. (2019). Transgenic Potato Lines Expressing Hairpin RNAi Construct of Molting-Associated *EcR* Gene Exhibit Enhanced Resistance against Colorado Potato Beetle (*Leptinotarsa Decemlineata*, *Say*). Transgenic. Res..

[60] Banakar P., Hada A., Papolu P.K., Rao U. (2020). Simultaneous RNAi Knockdown of Three FMRFamide-like Peptide Genes, *Mi-Flp1*, *Mi-Flp12*, and *Mi-Flp18* Provides Resistance to Root-Knot Nematode, *Meloidogyne incognita*. Front. Microbiol..

[61] Halder K., Chaudhuri A., Abdin M.Z., Majee M., Datta A. (2022). RNA Interference for Improving Disease Resistance in Plants and Its Relevance in This Clustered Regularly Interspaced Short Palindromic Repeats-Dominated Era in Terms of DsRNA-Based Biopesticides. Front. Plant Sci..

[62] Tenllado F., Díaz-Ruíz J.R. (2001). Double-Stranded RNA-Mediated Interference with Plant Virus Infection. J. Virol..

[63] McLoughlin A.G., Wytinck N., Walker P.L., Girard I.J., Rashid K.Y., De Kievit T., Fernando W.G.D., Whyard S., Belmonte M.F. (2018). Identification and Application of Exogenous DsRNA Confers Plant Protection against Sclerotinia Sclerotiorum and Botrytis Cinerea. Sci. Rep..

[64] Mitter N., Worrall E.A., Robinson K.E., Xu Z.P., Carroll B.J. (2017). Induction of Virus Resistance by Exogenous Application of Double-Stranded RNA. Curr. Opin. Virol..

[65] Höfle L., Biedenkopf D., Werner B.T., Shrestha A., Jelonek L., Koch A. (2020). Study on the Efficiency of DsRNAs with Increasing Length in RNA-Based Silencing of the *Fusarium CYP51* Genes. RNA Biol..

[66] Fletcher S.J., Reeves P.T., Hoang B.T., Mitter N. (2020). A Perspective on RNAi-Based Biopesticides. Front. Plant Sci..

[67] Kong X., Yang M., Le B.H., He W., Hou Y. (2022). The Master Role of SiRNAs in Plant Immunity. Mol. Plant Pathol..

[68] Wang X.B., Wu Q., Ito T., Cillo F., Li W.X., Chen X., Yu J.L., Ding S.W. (2010). RNAi-Mediated Viral Immunity Requires Amplification of Virus-Derived SiRNAs in Arabidopsis Thaliana. Proc. Natl. Acad. Sci. USA.

[69] Liu P., Zhang X., Zhang F., Xu M., Ye Z., Wang K., Liu S., Han X., Cheng Y., Zhong K. (2021). A Virus-Derived SiRNA Activates Plant Immunity by Interfering with ROS Scavenging. Mol. Plant.

[70] Cao M., Du P., Wang X., Yu Y.Q., Qiu Y.H., Li W., Gal-On A., Zhou C., Li Y., Ding S.W. (2014). Virus Infection Triggers Widespread Silencing of Host Genes by a Distinct Class of Endogenous SiRNAs in Arabidopsis. Proc. Natl. Acad. Sci. USA.

[71] Wagh S.G., Alam M.M., Kobayashi K., Yaeno T., Yamaoka N., Toriba T., Hirano H.Y., Nishiguchi M. (2016). Analysis of Rice *RNA-Dependent RNA Polymerase 6* (*OsRDR6*) Gene in Response to Viral, Bacterial and Fungal Pathogens. J. Gen. Plant Pathol..

[72] Nowara D., Schweizer P., Gay A., Lacomme C., Shaw J., Ridout C., Douchkov D., Hensel G., Kumlehn J. (2010). HIGS: Host-Induced Gene Silencing in the Obligate Biotrophic Fungal Pathogen Blumeria Graminis. Plant Cell.

[73] Regina A., Bird A., Topping D., Bowden S., Freeman J., Barsby T., Kosar-Hashemi B., Li Z., Rahman S., Morell M. (2006). High-Amylose Wheat Generated by RNA Interference Improves Indices of Large-Bowel Health in Rats. Proc. Natl. Acad. Sci. USA.

[74] Feldmann K.A. (2006). Steroid Regulation Improves Crop Yield. Nat. Biotechnol..

[75] Liu Q., Singh S.P., Green A.G. (2002). High-Stearic and High-Oleic Cottonseed Oils Produced by Hairpin RNA-Mediated Post-Transcriptional Gene Silencing. Plant Physiol..

[76] Eady C.C., Kamoi T., Kato M., Porter N.G., Davis S., Shaw M., Kamoi A., Imai S. (2008). Silencing Onion Lachrymatory Factor Synthase Causes a Significant Change in the Sulfur Secondary Metabolite Profile. Plant Physiol..

[77] Qiao F., Yang Q., Wang C.L., Fan Y.L., Wu X.F., Zhao K.J. (2007). Modification of Plant Height via RNAi Suppression of OsGA20ox2 Gene in Rice. Euphytica.

[78] Sunilkumar G., Campbell L.A.M., Puckhaber L., Stipanovic R.D., Rathore K.S. (2006). Engineering Cottonseed for Use in Human Nutrition by Tissue-Specific Reduction of Toxic Gossypol. Proc. Natl. Acad. Sci. USA.

[79] Jiang C.J., Shimono M., Maeda S., Inoue H., Mori M., Hasegawa M., Sugano S., Takatsuji H. (2009). Suppression of the Rice Fatty-Acid Desaturase Gene *OsSSI2* Enhances Resistance to Blast and Leaf Blight Diseases in Rice. Mol. Plant-Microbe Interact..

[80] Kusaba M., Miyahara K., Iida S., Fukuoka H., Takano T., Sassa H., Nishimura M., Nishio T. (2003). Low Glutelin Content1: A Dominant Mutation That Suppresses the Glutelin Multigene Family via RNA Silencing in Rice. Plant Cell.

[81] Yara A., Yaeno T., Hasegawa M., Seto H., Montillet J.L., Kusumi K., Seo S., Iba K. (2007). Disease Resistance against Magnaporthe Grisea Is Enhanced in Transgenic Rice with Suppression of ω-3 Fatty Acid Desaturases. Plant Cell Physiol..

[82] Sun L., Yuan B., Zhang M., Wang L., Cui M., Wang Q., Leng P. (2012). Fruit-Specific RNAi-Mediated Suppression of *SlNCED1* Increases Both Lycopene and β-Carotene Contents in Tomato Fruit. J. Exp. Bot..

[83] Eschen-Lippold L., Landgraf R., Smolka U., Schulze S., Heilmann M., Heilmann I., Hause G., Rosahl S. (2012). Activation of Defense against Phytophthora Infestans in Potato by Down-Regulation of Syntaxin Gene Expression. New Phytol..

[84] Davuluri G.R., Van Tuinen A., Fraser P.D., Manfredonia A., Newman R., Burgess D., Brummell D.A., King S.R., Palys J., Uhlig J. (2005). Fruit-Specific RNAi-Mediated Suppression of DET1 Enhances Carotenoid and Flavonoid Content in Tomatoes. Nat. Biotechnol..

[85] Riechen J. (2007). Establishment of Broad-Spectrum Resistance against *Blumeria Graminis f.Sp.* Tritici in *Triticum Aestivum* by RNAi-Mediated *Knock-down* of MLO. J. Für Verbrauch. Und Lebensm..

[86] Meli V.S., Ghosh S., Prabha T.N., Chakraborty N., Chakraborty S., Datta A. (2010). Enhancement of Fruit Shelf Life by Suppressing N-Glycan Processing Enzymes. Proc. Natl. Acad. Sci. USA.

[87] Huang G., Allen R., Davis E.L., Baum T.J., Hussey R.S. (2006). Engineering Broad Root-Knot Resistance in Transgenic Plants by RNAi Silencing of a Conserved and Essential Root-Knot Nematode Parasitism Gene. Proc. Natl. Acad. Sci. USA.

[88] Gupta A., Pal R.K., Rajam M.V. (2013). Delayed Ripening and Improved Fruit Processing Quality in Tomato by RNAi-Mediated Silencing of Three Homologs of 1-Aminopropane-1-Carboxylate Synthase Gene. J. Plant Physiol..

[89] Yadav B.C., Veluthambi K., Subramaniam K. (2006). Host-Generated Double Stranded RNA Induces RNAi in Plant-Parasitic Nematodes and Protects the Host from Infection. Mol. Biochem. Parasitol..

[90] Schijlen E.G.W.M., De Vos C.H.R., Martens S., Jonker H.H., Rosin F.M., Molthoff J.W., Tikunov Y.M., Angenent G.C., Van Tunen A.J., Bovy A.G. (2007). RNA Interference Silencing of Chalcone Synthase, the First Step in the Flavonoid Biosynthesis Pathway, Leads to Parthenocarpic Tomato Fruits. Plant Physiol..

[91] Shimizu T., Yoshii M., Wei T., Hirochika H., Omura T. (2009). Silencing by RNAi of the Gene for Pns12, a Viroplasm Matrix Protein of Rice Dwarf Virus, Results in Strong Resistance of Transgenic Rice Plants to the Virus. Plant Biotechnol. J..

[92] Niu Q.W., Lin S.S., Reyes J.L., Chen K.C., Wu H.W., Yeh S.D., Chua N.H. (2006). Expression of Artificial MicroRNAs in Transgenic Arabidopsis Thaliana Confers Virus Resistance. Nat. Biotechnol..

[93] Wang Y., Beaith M., Chalifoux M., Ying J., Uchacz T., Sarvas C., Griffiths R., Kuzma M., Wan J., Huang Y. (2009). Shoot-Specific down-Regulation of Protein Farnesyltransferase (α-Subunit) for Yield Protection against Drought in Canola. Mol. Plant.

[94] Wang T., Chen L., Zhao M., Tian Q., Zhang W.-H. (2011). Identification of Drought-Responsive MicroRNAs in Medicago Truncatula by Genome-Wide High- Throughput Sequencing. BMC Genom..

[95] Widyaningrum S., Pujiasih D.R., Sholeha W., Harmoko R., Sugiharto B. (2021). Induction of Resistance to Sugarcane Mosaic Virus by RNA Interference Targeting Coat Protein Gene Silencing in Transgenic Sugarcane. Mol. Biol. Rep..

[96] Miroshnichenko D., Timerbaev V., Okuneva A., Klementyeva A., Sidorova T., Pushin A., Dolgov S. (2020). Enhancement of Resistance to PVY in Intragenic Marker-Free Potato Plants by RNAi-Mediated Silencing of EIF4E Translation Initiation Factors. Plant Cell Tissue Organ. Cult..

[97] Ramesh S.V., Shivakumar M., Praveen S., Chouhan B.S., Chand S. (2019). Expression of Short Hairpin RNA (ShRNA) Targeting AC2 Gene of Mungbean Yellow Mosaic India Virus (MYMIV) Reduces the Viral Titre in Soybean. 3 Biotech.

[98] Kumari A., Hada A., Subramanyam K., Theboral J., Misra S., Ganapathi A., Malathi V.G. (2018). RNAi-Mediated Resistance to Yellow Mosaic Viruses in Soybean Targeting Coat Protein Gene. Acta Physiol. Plant.

[99] Yang X., Niu L., Zhang W., Yang J., Xing G., He H., Guo D., Du Q., Qian X., Yao Y. (2018). RNAi-Mediated SMV P3 Cistron Silencing Confers Significantly Enhanced Resistance to Multiple Potyvirus Strains and Isolates in Transgenic Soybean. Plant Cell Rep..

[100] Ahmed M.M.S., Bian S., Wang M., Zhao J., Zhang B., Liu Q., Zhang C., Tang S., Gu M., Yu H. (2017). RNAi-Mediated Resistance to Rice Black-Streaked Dwarf Virus in Transgenic Rice. Transgenic Res..

[101] Khatoon S., Kumar A., Sarin N.B., Khan J.A. (2016). RNAi-Mediated Resistance against Cotton Leaf Curl Disease in Elite Indian Cotton (Gossypium Hirsutum) Cultivar Narasimha. Virus Genes.

[102] Wang J., Mei J., Ren G. (2019). Plant MicroRNAs: Biogenesis, Homeostasis, and Degradation. Front. Plant Sci..

[103] Nozawa M., Miura S., Nei M. (2012). Origins and Evolution of MicroRNA Genes in Plant Species. Genome Biol. Evol..

[104] Budak H., Akpinar B.A. (2015). Plant MiRNAs: Biogenesis, Organization and Origins. Funct. Integr. Genom..

[105] Rogers K., Chen X. (2013). Biogenesis, Turnover, and Mode of Action of Plant MicroRNAs. Plant Cell.

[106] Dong Z., Han M.H., Fedoroff N. (2008). The RNA-Binding Proteins HYL1 and SE Promote Accurate in Vitro Processing of Pri-MiRNA by DCL1. Proc. Natl. Acad. Sci. USA.

[107] Wu L., Zhou H., Zhang Q., Zhang J., Ni F., Liu C., Qi Y. (2010). DNA Methylation Mediated by a MicroRNA Pathway. Mol. Cell.

[108] Francisco-Mangilet A.G., Karlsson P., Kim M.H., Eo H.J., Oh S.A., Kim J.H., Kulcheski F.R., Park S.K., Manavella P.A. (2015). THO2, a Core Member of the THO/TREX Complex, Is Required for MicroRNA Production in Arabidopsis. Plant J..

[109] Karlsson P., Christie M.D., Seymour D.K., Wang H., Wang X., Hagmann J., Kulcheski F., Manavella P.A. (2015). KH Domain Protein RCF3 Is a Tissue-Biased Regulator of the Plant MiRNA Biogenesis Cofactor HYL1. Proc. Natl. Acad. Sci. USA.

[110] Wang L., Song X., Gu L., Li X., Cao S., Chu C., Cui X., Chen X., Cao X. (2013). NOT2 Proteins Promote Polymerase II—Dependent Transcription and Interact with Multiple MicroRNA Biogenesis Factors in Arabidopsis. Plant Cell.

[111] Wu X., Shi Y., Li J., Xu L., Fang Y., Li X., Qi Y. (2013). A Role for the RNA-Binding Protein MOS2 in MicroRNA Maturation in Arabidopsis. Cell Res..

[112] Qiao Y., Shi J., Zhai Y., Hou Y., Ma W. (2015). Phytophthora Effector Targets a Novel Component of Small RNA Pathway in Plants to Promote Infection. Proc. Natl. Acad. Sci. USA.

[113] Fang X., Cui Y., Li Y., Qi Y. (2015). Transcription and Processing of Primary MicroRNAs Are Coupled by Elongator Complex in Arabidopsis. Nat. Plants.

[114] Zhang S., Xie M., Ren G., Yu B. (2013). CDC5, a DNA Binding Protein, Positively Regulates Posttranscriptional Processing and / or Transcription of Primary MicroRNA Transcripts. Proc. Natl. Acad. Sci. USA.

[115] Bologna N.G., Voinnet O. (2014). The Diversity, Biogenesis, and Activities of Endogenous Silencing Small RNAs in Arabidopsis. Annu. Rev. Plant Biol..

[116] Addo-Quaye C., Snyder J.A., Park Y.B., Li Y.F., Sunkar R., Axtell M.J. (2009). Sliced MicroRNA Targets and Precise Loop-First Processing of MIR319 Hairpins Revealed by Analysis of the Physcomitrella Patens Degradome. RNA.

[117] Bologna N.G., Mateos J.L., Bresso E.G., Palatnik J.F. (2009). A Loop-to-Base Processing Mechanism Underlies the Biogenesis of Plant MicroRNAs MiR319 and MiR159. EMBO J..

[118] Bologna N.G., Schapire A.L., Zhai J., Chorostecki U., Boisbouvier J., Meyers B.C., Palatnik J.F. (2013). Multiple RNA Recognition Patterns during MicroRNA Biogenesis in Plants. Genome Res..

[119] Mateos J.L., Bologna N.G., Chorostecki U., Palatnik J.F. (2010). Identification of MicroRNA Processing Determinants by Random Mutagenesis of Arabidopsis MIR172a Precursor. Curr. Biol..

[120] Song L., Axtell M.J., Fedoroff N.V. (2010). RNA Secondary Structural Determinants of MiRNA Precursor Processing in Arabidopsis. Curr. Biol..

[121] Werner S., Wollmann H., Schneeberger K., Weigel D. (2010). Structure Determinants for Accurate Processing of MiR172a in Arabidopsis Thaliana. Curr. Biol..

[122] Yu B., Yang Z., Li J., Minakhina S., Yang M., Padgett R.W., Steward R., Chen X. (2005). Methylation as a Crucial Step in Plant MicroRNA Biogenesis Bin. Science.

[123] Yang Z., Ebright Y.W., Yu B., Chen X. (2006). HEN1 Recognizes 21-24 Nt Small RNA Duplexes and Deposits a Methyl Group onto the 2′ OH of the 3′ Terminal Nucleotide. Nucleic Acids Res..

[124] Mee Y.P., Wu G., Gonzalez-Sulser A., Vaucheret H., Poethig R.S. (2005). Nuclear Processing and Export of MicroRNAs in Arabidopsis. Proc. Natl. Acad. Sci. USA.

[125] Zhang H., Xia R., Meyers B.C., Walbot V. (2015). Evolution, Functions, and Mysteries of Plant ARGONAUTE Proteins. Curr. Opin. Plant Biol..

[126] Wang H., Wang H., Duan X., Liu C., Li Z. (2017). Digital Quantitative Analysis of MicroRNA in Single Cell Based on Ligation-Depended Polymerase Colony (Polony). Biosens. Bioelectron..

[127] Carthew R.W., Sontheimer E.J. (2009). Origins and Mechanisms of MiRNAs and SiRNAs. Cell.

[128] Sunkar R., Zhu J.K. (2007). Micro RNAs and Short-Interfering RNAs in Plants. J. Integr. Plant Biol..

[129] Kim V.N. (2005). Small RNAs: Classification, Biogenesis, and Function. Mol. Cells.

[130] Zilberman D., Cao X., Jacobsen S.E. (2003). ARGONAUTE4 Control of Locus-Specific SiRNA Accumulation and DNA and Histone Methylation. Science.

[131] Kim V.N., Han J., Siomi M.C. (2009). Biogenesis of Small RNAs in Animals. Nat. Rev. Mol. Cell Biol..

[132] Watanabe T., Totoki Y., Sasaki H., Minami N., Imai H. (2007). Analysis of Small RNA Profiles During Development. Methods Enzymol..

[133] Velasco R., Zharkikh A., Troggio M., Cartwright D.A., Cestaro A., Pruss D., Pindo M., FitzGerald L.M., Vezzulli S., Reid J. (2007). A High Quality Draft Consensus Sequence of the Genome of a Heterozygous Grapevine Variety. PLoS ONE.

[134] Fei Q., Xia R., Meyers B.C. (2013). Phased, Secondary, Small Interfering RNAs in Posttranscriptional Regulatory Networks. Plant Cell.

[135] Creasey K.M., Zhai J., Borges F., Van Ex F., Regulski M., Meyers B.C., Martienssen R.A. (2014). MiRNAs Trigger Widespread Epigenetically-Activated SiRNAs from Transposons in Arabidopsis. Nature.

[136] Deng P., Muhammad S., Cao M., Wu L. (2018). Biogenesis and Regulatory Hierarchy of Phased Small Interfering RNAs in Plants. Plant Biotechnol. J..

[137] Schwab R., Palatnik J.F., Riester M., Schommer C., Schmid M., Weigel D. (2005). Specific Effects of MicroRNAs on the Plant Transcriptome. Dev. Cell.

[138] Axtell M.J., Meyers B.C. (2018). Revisiting Criteria for Plant MicroRNA Annotation in the Era of Big Data. Plant Cell.

[139] Carbonell A., Fahlgren N., Garcia-Ruiz H., Gilbert K.B., Montgomery T.A., Nguyen T., Cuperus J.T., Carrington J.C. (2012). Functional Analysis of Three Arabidopsis Argonautes Using Slicer-Defective Mutants. Plant Cell.

[140] Köhler A., Hurt E. (2007). Exporting RNA from the Nucleus to the Cytoplasm. Nat. Rev. Mol. Cell Biol..

[141] Chen X., Sciences P., States U. (2005). microRNA biogenesis and function in plants. FEBS Lett..

[142] Chen X. (2009). Small RNAs and Their Roles in Plant Development. Annu. Rev. Cell Dev. Biol..

[143] Hutva’gner G., Zamore P.D. (2002). A MicroRNA in a Multiple- Turnover RNAi Enzyme Complex. Science.

[144] Brodersen P., Sakvarelidze-Achard L., Bruun-Rasmussen M., Dunoyer P., Yamamoto Y.Y., Sieburth L., Voinnet O. (2008). Widespread Translational Inhibition by Plant MiRNAs and SiRNAs. Science.

[145] Yang L., Wu G., Poethig R.S. (2012). Mutations in the GW-Repeat Protein SUO Reveal a Developmental Function for MicroRNA-Mediated Translational Repression in Arabidopsis. Proc. Natl. Acad. Sci. USA.

[146] Li S., Liu L., Zhuang X., Yu Y., Liu X., Cui X., Ji L., Pan Z., Cao X., Mo B. (2013). MicroRNAs Inhibit the Translation of Target MRNAs on the Endoplasmic Reticulum in Arabidopsis. Cell.

[147] Aukerman M.J., Sakai H. (2003). Regulation of Flowering Time and Floral Organ Identity by a MicroRNA and Its *APETALA2*-like Target Genes The Plant Cell. Plant Cell.

[148] Gandikota M., Birkenbihl R.P., Höhmann S., Cardon G.H., Saedler H., Huijser P. (2007). The MiRNA156/157 Recognition Element in the 3′ UTR of the Arabidopsis SBP Box Gene SPL3 Prevents Early Flowering by Translational Inhibition in Seedlings. Plant J..

[149] Chen X. (2004). A MicroRNA as a Translational Repressor of APETALA2 in Arabidopsis Flower Development. Science.

[150] Hou C.Y., Lee W.C., Chou H.C., Chen A.P., Chou S.J., Chen H.M. (2016). Global Analysis of Truncated RNA Ends Reveals New Insights into Ribosome Stalling in Plants. Plant Cell.

[151] Li S., Le B., Ma X., Li S., You C., Yu Y., Zhang B., Liu L., Gao L., Shi T. (2016). Biogenesis of Phased SiRNAs on Membrane-Bound Polysomes in Arabidopsis. Elife.

[152] Yu X., Willmann M.R., Anderson S.J., Gregory B.D. (2016). Genome-Wide Mapping of Uncapped and Cleaved Transcripts Reveals a Role for the Nuclear Mrna Cap-Binding Complex in Cotranslational Rna Decay in Arabidopsis. Plant Cell.

[153] Kamthan A., Chaudhuri A., Kamthan M., Datta A. (2015). Small RNAs in Plants: Recent Development and Application for Crop Improvement. Front. Plant Sci..

[154] Llave C., Xie Z., Kasschau K.D., Carrington J.C. (2002). Cleavage of Scarecrow-like MRNA Targets Directed by a Class of Arabidopsis MiRNA. Science.

[155] German M.A., Pillay M., Jeong D.H., Hetawal A., Luo S., Janardhanan P., Kannan V., Rymarquis L.A., Nobuta K., German R. (2008). Global Identification of MicroRNA-Target RNA Pairs by Parallel Analysis of RNA Ends. Nat. Biotechnol..

[156] Mi S., Cai T., Hu Y., Chen Y., Hodges E., Ni F., Wu L., Li S., Zhou H., Long C. (2008). Sorting of Small RNAs into Arabidopsis Argonaute Complexes Is Directed by the 5′ Terminal Nucleotide. Cell.

[157] Montgomery T.A., Howell M.D., Cuperus J.T., Li D., Hansen J.E., Alexander A.L., Chapman E.J., Fahlgren N., Allen E., Carrington J.C. (2008). Specificity of ARGONAUTE7-MiR390 Interaction and Dual Functionality in TAS3 Trans-Acting SiRNA Formation. Cell.

[158] Takeda A., Iwasaki S., Watanabe T., Utsumi M., Watanabe Y. (2008). The Mechanism Selecting the Guide Strand from Small RNA Duplexes Is Different among Argonaute Proteins. Plant Cell Physiol..

[159] Ji L., Liu X., Yan J., Wang W., Yumul R.E., Kim Y.J., Dinh T.T., Liu J., Cui X., Zheng B. (2011). ARGONAUTE10 and ARGONAUTE1 Regulate the Termination of Floral Stem Cells through Two MicroRNAs in Arabidopsis. PLoS Genet..

[160] Maunoury N., Vaucheret H. (2011). AGO1 and AGO2 Act Redundantly in MiR408-Mediated Plantacyanin Regulation. PLoS ONE.

[161] Zhu H., Hu F., Wang R., Zhou X., Sze S.H., Liou L.W., Barefoot A., Dickman M., Zhang X. (2011). Arabidopsis Argonaute10 Specifically Sequesters MiR166/165 to Regulate Shoot Apical Meristem Development. Cell.

[162] Souret F.F., Kastenmayer J.P., Green P.J. (2004). AtXRN4 Degrades MRNA in Arabidopsis and Its Substrates Include Selected MiRNA Targets. Mol. Cell.

[163] Ibrahim F., Rohr J., Jeong W.J., Hesson J., Cerutti H. (2006). Untemplated Oligoadenylation Promotes Degradation of RISC-Cleaved Transcripts. Science.

[164] Ren G., Xie M., Zhang S., Vinovskis C., Chen X., Yu B. (2014). Methylation Protects MicroRNAs from an AGO1-Associated Activity That Uridylates 5′ RNA Fragments Generated by AGO1 Cleavage. Proc. Natl. Acad. Sci. USA.

[165] Wang X., Zhang S., Dou Y., Zhang C., Chen X., Yu B., Ren G. (2015). Synergistic and Independent Actions of Multiple Terminal Nucleotidyl Transferases in the 3′ Tailing of Small RNAs in Arabidopsis. PLoS Genet..

[166] Zhang Z., Hu F., Sung M.W., Shu C., Castillo-González C., Koiwa H., Tang G., Dickman M., Li P., Zhang X. (2017). RISC-Interacting Clearing 3′-5′ Exoribonucleases (RICES) Degrade Uridylated Cleavage Fragments to Maintain Functional RISC in Arabidopsis Thaliana. Elife.

[167] Branscheid A., Marchais A., Schott G., Lange H., Gagliardi D., Andersen S.U., Voinnet O., Brodersen P. (2015). SKI2 Mediates Degradation of RISC 5′-Cleavage Fragments and Prevents Secondary SiRNA Production from MiRNA Targets in Arabidopsis. Nucleic Acids Res..

[168] Iwakawa H.O., Tomari Y. (2013). Molecular Insights into MicroRNA-Mediated Translational Repression in Plants. Mol Cell.

[169] Yu Y., Jia T., Chen X. (2017). The ‘How’ and ‘Where’ of Plant MicroRNAs. New Phytol..

[170] Zhang X., Zou Z., Gong P., Zhang J., Ziaf K., Li H., Xiao F., Ye Z. (2011). Over-Expression of MicroRNA169 Confers Enhanced Drought Tolerance to Tomato. Biotechnol. Lett..

[171] Li C., Zhang B. (2016). MicroRNAs in Control of Plant Development. J. Cell Physiol..

[172] Swarup R., Denyer T. (2019). MiRNAs in Plant Development. Annual Plant Reviews Online.

[173] Yu Y., Zhang Y., Chen X., Chen Y. (2019). Plant Noncoding RNAs: Hidden Players in Development and Stress Responses. Annu. Rev. Cell Dev. Biol..

[174] Ma J., Zhao P., Liu S., Yang Q., Guo H. (2020). The Control of Developmental Phase Transitions by MicroRNAs and Their Targets in Seed Plants. Int. J. Mol. Sci..

[175] Bhogireddy S., Mangrauthia S.K., Kumar R., Pandey A.K., Singh S., Jain A., Budak H., Varshney R.K., Kudapa H. (2021). Regulatory Non-Coding RNAs: A New Frontier in Regulation of Plant Biology. Funct. Integr. Genom..

[176] Liu Y., El-Kassaby Y.A. (2017). Regulatory Crosstalk between MicroRNAs and Hormone Signalling Cascades Controls the Variation on Seed Dormancy Phenotype at Arabidopsis Thaliana Seed Set. Plant Cell Rep..

[177] Das S.S., Karmakar P., Nandi A.K., Sanan-Mishra N. (2015). Small RNA Mediated Regulation of Seed Germination. Front. Plant Sci..

[178] Martin R.C., Asahina M., Liu P.P., Kristof J.R., Coppersmith J.L., Pluskota W.E., Bassel G.W., Goloviznina N.A., Nguyen T.T., Martínez-Andújar C. (2010). The MicroRNA156 and MicroRNA172 Gene Regulation Cascades at Post-Germinative Stages in Arabidopsis. Seed Sci. Res..

[179] Alptekin B., Langridge P., Budak H. (2017). Abiotic Stress MiRNomes in the Triticeae. Funct. Integr. Genom..

[180] Song X., Li Y., Cao X., Qi Y. (2019). MicroRNAs and Their Regulatory Roles in Plant-Environment Interactions. Annu. Rev. Plant Biol..

[181] Ferdous J., Sanchez-Ferrero J.C., Langridge P., Milne L., Chowdhury J., Brien C., Tricker P.J. (2017). Differential Expression of MicroRNAs and Potential Targets under Drought Stress in Barley. Plant Cell Environ..

[182] Li W.X., Oono Y., Zhu J., He X.J., Wu J.M., Iida K., Lu X.Y., Cui X., Jin H., Zhu J.K. (2008). The Arabidopsis NFYA5 Transcription Factor Is Regulated Transcriptionally and Posttranscriptionally to Promote Drought Resistance. Plant Cell.

[183] Guan Q., Lu X., Zeng H., Zhang Y., Zhu J. (2013). Heat Stress Induction of MiR398 Triggers a Regulatory Loop That Is Critical for Thermotolerance in Arabidopsis. Plant J..

[184] Song J.B., Gao S., Wang Y., Li B.W., Zhang Y.L., Yang Z.M. (2016). MiR394 and Its Target Gene LCR Are Involved in Cold Stress Response in Arabidopsis. Plant Gene.

[185] Zhou M., Li D., Li Z., Hu Q., Yang C., Zhu L., Luo H. (2013). Constitutive Expression of a MiR319 Gene Alters Plant Development and Enhances Salt and Drought Tolerance in Transgenic Creeping Bentgrass. Plant Physiol..

[186] Salvador-Guirao R., Baldrich P., Weigel D., Segundo B.S., Rubio-Somoza I. (2018). The Microrna MiR773 Is Involved in the Arabidopsis Immune Response to Fungal Pathogens. Mol. Plant-Microbe Interact..

[187] Gao Y., Li S.J., Zhang S.W., Feng T., Zhang Z.Y., Luo S.J., Mao H.Y., Borkovich K.A., Ouyang S.Q. (2021). SlymiR482e-3p Mediates Tomato Wilt Disease by Modulating Ethylene Response Pathway. Plant Biotechnol. J..

[188] Canto-Pastor A., Santos B.A.M.C., Valli A.A., Summers W., Schornack S., Baulcombe D.C. (2019). Enhanced Resistance to Bacterial and Oomycete Pathogens by Short Tandem Target Mimic RNAs in Tomato. Proc. Natl. Acad. Sci. USA.

[189] Hewezi T., Piya S., Qi M., Balasubramaniam M., Rice J.H., Baum T.J. (2016). Arabidopsis MiR827 Mediates Post-Transcriptional Gene Silencing of Its Ubiquitin E3 Ligase Target Gene in the Syncytium of the Cyst Nematode Heterodera Schachtii to Enhance Susceptibility. Plant J..

[190] Wamiq G., Khan J.A. (2018). Overexpression of Ghr-MiR166b Generates Resistance against Bemisia Tabaci Infestation in Gossypium Hirsutum Plants. Planta.

[191] Song Z., Zhang L., Wang Y., Li H., Li S., Zhao H., Zhang H. (2018). Constitutive Expression of Mir408 Improves Biomass and Seed Yield in Arabidopsis. Front. Plant Sci..

[192] Gao F., Wang K., Liu Y., Chen Y., Chen P., Shi Z., Luo J., Jiang D., Fan F., Zhu Y. (2016). Blocking MiR396 Increases Rice Yield by Shaping Inflorescence Architecture. Nat. Plants.

[193] Schommer C., Palatnik J.F., Aggarwal P., Chételat A., Cubas P., Farmer E.E., Nath U., Weigel D. (2008). Control of Jasmonate Biosynthesis and Senescence by MiR319 Targets. PLoS Biol..

[194] Ozseyhan M.E., Li P., Na G.N., Li Z., Wang C., Lu C. (2018). Improved Fatty Acid Profiles in Seeds of Camelina Sativa by Artificial MicroRNA Mediated FATB Gene Suppression. Biochem. Biophys. Res. Commun..

[195] Zhang H., Chen H., Hou Z., Xu L., Jin W., Liang Z. (2020). Overexpression of Ath-MIR160b Increased the Biomass While Reduced the Content of Tanshinones in Salvia Miltiorrhiza Hairy Roots by Targeting ARFs Genes. Plant Cell Tissue Organ. Cult..

[196] Jia X., Shen J., Liu H., Li F., Ding N., Gao C., Pattanaik S., Patra B., Li R., Yuan L. (2015). Small Tandem Target Mimic-Mediated Blockage of MicroRNA858 Induces Anthocyanin Accumulation in Tomato. Planta.

[197] Sunkar R., Kapoor A., Zhu J.K. (2006). Erratum: Posttranscriptional Induction of Two Cu/Zn Superoxide Dismutase Genes in Arabidopsis Is Mediated by Downregulation of MiR398 and Important for Oxidative Stress. Plant Cell.

[198] Bai Q., Wang X., Chen X., Shi G., Liu Z., Guo C., Xiao K. (2018). Wheat MiRNA Taemir408 Acts as an Essential Mediator in Plant Tolerance to Pi Deprivation and Salt Stress via Modulating Stress-Associated Physiological Processes. Front. Plant Sci..

[199] Yan Y., Wang H., Hamera S., Chen X., Fang R. (2014). MiR444a Has Multiple Functions in the Rice Nitrate-Signaling Pathway. Plant J..

[200] Gao S., Guo C., Zhang Y., Zhang F., Du X., Gu J., Xiao K. (2016). Wheat MicroRNA Member TaMIR444a Is Nitrogen Deprivation-Responsive and Involves Plant Adaptation to the Nitrogen-Starvation Stress. Plant Mol. Biol. Rep..

[201] Wu J., Yang R., Yang Z., Yao S., Zhao S., Wang Y., Li P., Song X., Jin L., Zhou T. (2017). ROS Accumulation and Antiviral Defence Control by MicroRNA528 in Rice. Nat. Plants.

[202] Wang H., Jiao X., Kong X., Hamera S., Wu Y., Chen X., Fang R., Yan Y. (2016). A Signaling Cascade from MiR444 to RDR1 in Rice Antiviral RNA Silencing Pathway. Plant Physiol..

[203] Mao Y.B., Liu Y.Q., Chen D.Y., Chen F.Y., Fang X., Hong G.J., Wang L.J., Wang J.W., Chen X.Y. (2017). Jasmonate Response Decay and Defense Metabolite Accumulation Contributes to Age-Regulated Dynamics of Plant Insect Resistance. Nat. Commun..

[204] Soto-Suárez M., Baldrich P., Weigel D., Rubio-Somoza I., San Segundo B. (2017). The Arabidopsis MiR396 Mediates Pathogen-Associated Molecular Pattern-Triggered Immune Responses against Fungal Pathogens. Sci. Rep..

[205] Dai Z., Tan J., Zhou C., Yang X., Yang F., Zhang S., Sun S., Miao X., Shi Z. (2019). The OsmiR396–OsGRF8–OsF3H-Flavonoid Pathway Mediates Resistance to the Brown Planthopper in Rice (*Oryza sativa*). Plant Biotechnol. J..

[206] Zubair M., Khan M.Z., Rauf I., Raza A., Shah A.H., Hassan I., Amin I., Mansoor S. (2020). Artificial Micro RNA (AmiRNA)-Mediated Resistance against Whitefly (*Bemisia tabaci*) Targeting Three Genes. Crop Prot..

[207] Feyissa B.A., Arshad M., Gruber M.Y., Kohalmi S.E., Hannoufa A. (2019). The Interplay between MiR156/SPL13 and DFR/WD40-1 Regulate Drought Tolerance in Alfalfa. BMC Plant Biol..

[208] Visentin I., Pagliarani C., Deva E., Caracci A., Turečková V., Novák O., Lovisolo C., Schubert A., Cardinale F. (2020). A Novel Strigolactone-MiR156 Module Controls Stomatal Behaviour during Drought Recovery. Plant Cell Environ..

[209] Kim J.Y., Kwak K.J., Jung H.J., Lee H.J., Kang H. (2010). MicroRNA402 Affects Seed Germination of Arabidopsis Thaliana under Stress Conditions via Targeting DEMETER-LIKE Protein3 MRNA. Plant Cell Physiol..

[210] Lin J.S., Kuo C.C., Yang I.C., Tsai W.A., Shen Y.H., Lin C.C., Liang Y.C., Li Y.C., Kuo Y.W., King Y.C. (2018). MicroRNA160 Modulates Plant Development and Heat Shock Protein Gene Expression to Mediate Heat Tolerance in Arabidopsis. Front. Plant Sci..

[211] Giacomelli J.I., Weigel D., Chan R.L., Manavella P.A. (2012). Role of Recently Evolved MiRNA Regulation of Sunflower HaWRKY6 in Response to Temperature Damage. New Phytol..

[212] Ding Y., Gong S., Wang Y., Wang F., Bao H., Sun J., Cai C., Yi K., Chen Z., Zhu C. (2018). MicroRNA166 Modulates Cadmium Tolerance and Accumulation in Rice. Plant Physiol..

[213] Zhao Y., Xu K., Liu G., Li S., Zhao S., Liu X., Yang X., Xiao K. (2020). Global Identification and Characterization of MiRNA Family Members Responsive to Potassium Deprivation in Wheat (*Triticum aestivum* L.). Sci. Rep..

[214] Peragine A., Yoshikawa M., Wu G., Albrecht H.L., Poethig R.S. (2004). SGS3 and SGS2/SDE1/RDR6 Are Required for Juvenile Development and the Production of Trans-Acting SiRNAs in Arabidopsis. Genes Dev..

[215] Vazquez F., Vaucheret H., Rajagopalan R., Lepers C., Gasciolli V., Mallory A.C., Hilbert J.L., Bartel D.P., Crété P. (2004). Endogenous Trans-Acting SiRNAs Regulate the Accumulation of Arabidopsis MRNAs. Mol. Cell.

[216] Allen E., Xie Z., Gustafson A.M., Carrington J.C. (2005). MicroRNA-Directed Phasing during Trans-Acting SiRNA Biogenesis in Plants. Cell.

[217] Williams L., Carles C.C., Osmont K.S., Fletcher J.C. (2005). A Database Analysis Method Identifies an Endogenous Trans-Acting Short-Interfering RNA That Targets the *Arabidopsis ARF2*, *ARF3*, and *ARF4* Genes. Proc. Natl. Acad. Sci. USA.

[218] Rajagopalan R., Vaucheret H., Trejo J., Bartel D.P. (2006). A Diverse and Evolutionarily Fluid Set of MicroRNAs in Arabidopsis Thaliana. Genes Dev..

[219] Arif M.A., Fattash I., Ma Z., Cho S.H., Beike A.K., Reski R., Axtell M.J., Frank W. (2012). DICER-LIKE3 Activity in Physcomitrella Patens DICER-LIKE4 Mutants Causes Severe Developmental Dysfunction and Sterility. Mol. Plant.

[220] Zhang C., Li G., Wang J., Fang J. (2012). Identification of Trans-Acting SiRNAs and Their Regulatory Cascades in Grapevine. Bioinformatics.

[221] Li F., Orban R., Baker B. (2012). SoMART: A Web Server for Plant MiRNA, TasiRNA and Target Gene Analysis. Plant J..

[222] Zuo J., Wang Q., Han C., Ju Z., Cao D., Zhu B., Luo Y., Gao L. (2017). SRNAome and Degradome Sequencing Analysis Reveals Specific Regulation of SRNA in Response to Chilling Injury in Tomato Fruit. Physiol. Plant.

[223] Wu L., Mao L., Qi Y. (2012). Roles of DICER-LIKE and ARGONAUTE Proteins in TAS-Derived Small Interfering RNA-Triggered DNA Methylation. Plant Physiol..

[224] Montgomery T.A., Seong J.Y., Fahlgren N., Gilbert S.D., Howell M.D., Sullivan C.M., Alexander A., Nguyen G., Allen E., Ji H.A. (2008). AGO1-MiR173 Complex Initiates Phased SiRNA Formation in Plants. Proc. Natl. Acad. Sci. USA.

[225] Cuperus J.T., Carbonell A., Fahlgren N., Garcia-Ruiz H., Burke R.T., Takeda A., Sullivan C.M., Gilbert S.D., Montgomery T.A., Carrington J.C. (2010). Unique Functionality of 22-Nt MiRNAs in Triggering RDR6-Dependent SiRNA Biogenesis from Target Transcripts in Arabidopsis. Nat. Struct. Mol. Biol..

[226] Sanan-Mishra N., Abdul Kader Jailani A., Mandal B., Mukherjee S.K. (2021). Secondary SiRNAs in Plants: Biosynthesis, Various Functions, and Applications in Virology. Front. Plant Sci..

[227] Li S., Liu J., Liu Z., Li X., Wu F., He Y. (2014). HEAT-INDUCED TAS1 TARGET1 Mediates Thermotolerance via Heat Stress Transcription Factor A1a-Directed Pathways in Arabidopsis. Plant Cell.

[228] Kume K., Tsutsumi K.I., Saitoh Y. (2010). TAS1 Trans-Acting SiRNA Targets Are Differentially Regulated at Low Temperature, and TAS1 Trans-Acting SiRNA Mediates Temperature-Controlled At1g51670 Expression. Biosci. Biotechnol. Biochem..

[229] Chitwood D.H., Nogueira F.T.S., Howell M.D., Montgomery T.A., Carrington J.C., Timmermans M.C.P. (2009). Pattern Formation via Small RNA Mobility. Genes Dev..

[230] Xia J., Wang X., Perroud P.F., He Y., Quatrano R., Zhang W. (2016). Endogenous Small-Noncoding RNAs and Potential Functions in Desiccation Tolerance in Physcomitrella Patens. Sci. Rep..

[231] Shi M.-Z., Xie D.-Y. (2014). Biosynthesis and Metabolic Engineering of Anthocyanins in Arabidopsis Thaliana. Recent Pat. Biotechnol..

[232] Zheng Y., Wang Y., Wu J., Ding B., Fei Z. (2015). A Dynamic Evolutionary and Functional Landscape of Plant Phased Small Interfering RNAs. BMC Biol..

[233] Khraiwesh B., Zhu J.K., Zhu J. (2012). Role of MiRNAs and SiRNAs in Biotic and Abiotic Stress Responses of Plants. Biochim. Biophys. Acta Gene Regul. Mech..

[234] Zhou B., Leng J., Ma Y., Fan P., Li Y., Yan H., Xu Q. (2020). BrmiR828 Targets BrPAP1, BrMYB82, and BrTAS4 Involved in the Light Induced Anthocyanin Biosynthetic Pathway in Brassica Rapa. Int. J. Mol. Sci..

[235] Cai Q., Qiao L., Wang M., He B., Lin F.M., Palmquist J., Huang S.D., Jin H. (2018). Plants Send Small RNAs in Extracellular Vesicles to Fungal Pathogen to Silence Virulence Genes. Science.

[236] Ji N., van Oudenaarden A. (2012). Single Molecule Fluorescent in Situ Hybridization (SmFISH) of *C. elegans* Worms and Embryos. WormBook.

[237] Wu H., Yang L., Chen L.L. (2017). The Diversity of Long Noncoding RNAs and Their Generation. Trends Genet..

